# Supplementary material for: PRMT5 regulates alternative splicing of TCF3 under hypoxia to promote EMT and invasion in breast cancer
Source: PLoS Biol. 2025 Oct 28;23(10):e3003444. doi: 10.1371/journal.pbio.3003444 (PMC12585103; doi:10.1371/journal.pbio.3003444)
Supplement: S1 Table — (DOCX) [file pbio.3003444.s001.docx]

**S1 Table: List of primers**

| **Sno** | **Primers** | **Sequence (5’-3’)** |
| --- | --- | --- |
| 1 | PRMT5 Fwd | CCTTATGTGGTACGGCTGCAC |
| 2 | PRMT5 Rev | AGTACTGTGTTCACCTCCACAGG |
| 3 | PRMT5_ pro_CTCFbs_Fwd | CTATGGCTCTGTCCCCAACGA |
| 4 | PRMT5_pro_CTCFbs_Rev | CCTAGGATTCTGTCCATGCAGTGA |
| 5 | SgCTCFbs Fwd | CACCGAAAGAGGGTTTGGGAAATCA |
| 6 | SgCTCFbs Rev | AAACTGATTTCCCAAACCCTCTTTC |
| 7 | TCF3_ICR_F | GCG GCT GCT TCC TTA ACT CC |
| 8 | TCF3_ICR_R | GCTGAGGGGATAGCGTGTGG |
| 9 | PRMT5_ProSDM_F | CCTCCATCCAACTTTCTATTACAAAGAACTTC  ATATATGTACAATTCAGCATGACCAAACCCAA ACCCTGATTTC |
| 10 | PRMT5_ProSDM_R | GAAATCAGGGTTTGGGTTTGGTCATGCTGAA  TTGTACATATATGAAGTTCTTTGTAATAGAAA GTTGGATGGAGG |
| 11 | dCAS9-PRMT5_Fwd | CGG ATC CAT GGC GGC GAT GGC GGT CG |
| 12 | dCAS9-PRMT5_Rev | ACGCGTCGACGTAGAGGCCAATGGTATATGA GC |
| 13 | TCF3_Cons_Fwd | ACTCCTACAGTGGGCTAGGG |
| 14 | TCF3_Cons_Rev | TCTTCTCCTCCTCCGAGTGG |
| 15 | TCF3_18B_Fwd | CCGGACCAGCAGTACGG |
| 16 | TCF3_18B_Rev | GCCCCAGGATGACCTGC |
| 17 | TCF3_18A_Fwd | CCCAGACGAGGACGAGG |
| 18 | TCF3_18A_Rev | GCTGCTTTGGGATTCAGGTTCCG |
| 19 | PRMT5 promoter Rev | CCCAAGCTTGTGTCAGCTATTTCGGGGAC |
| 20 | PRMT5 -2000 Fwd | CGGGGTACCGAGACGGAGTCTTGCTGTTGCC  C |
| 21 | PRMT5 -1500 Fwd | CGGGGTACCGCCAGGCTGGTCTCGAACTC |
| 22 | PRMT5 -1000 Fwd | CGGGGTACCCCTTGCTTCCTTCCTTTCCATC |
| 23 | PRMT5 -500 Fwd | CGGGGTACCGACATTACATGGCTGCATAACC |
| 24 | PRMT5_OE_Fwd | CGCGGATCCGCGATGGCGGCGATGGCGGTC GG |
| 25 | PRMT5_OE_Rev | CCGCTCGAGCGGCTAGAGGCCAATGGTATAT GAGCG |
| 26 | sgTCF3-ICR_Fwd | CACCG CTCTGCTGTCTGCCTTCCGC |
| 27 | SgTCF3-ICR_Rev | AAACGCGGAAGGCAGACAGCAGAGC |
| 28 | TCF3_OE_Fwd | CGCGGATCCATGAACCAGCCGCAGAGG |
| 29 | TCF3_OE_Rev | CCCAAGCTTTCACATGTGCCCGGCGG |
| 30 | 3tag1A_MCS_Fwd | GATCCCCGGGCTGCAGGAATTCGATATCAAG  CTTATCGATACCGTCGACCTCGACCGG |
| 31 | 3tag1A_MCS_Rev | TCGAGGTCGACGGTATCGATAAGCTTGATATC GAATTCCTGCAGCCCGGG |
| 32 | sg1_ICRedit_Fwd | CACCGCTTCCCCCCGCTGGCGCTGG |
| 33 | sg1_ICRedit_Rev | AAACCAGCGCCAGCGGGGGGAAGC |
| 34 | sg2_ICRedit_Fwd | CACCG CTCTGCTGTCTGCCTTCCGC |
| 35 | sg2_ICRedit_Rev | AAACGCGGAAGGCAGACAGCAGAGC |
| 36 | Indel_analysis_Fwd | CCATAGCCCAGACGAGGAC |
| 37 | Indel_analysis_Rev | GCTCTAGGTTGTGGTGAAGATG |
| 38 | CDH1_Fwd | CTGAGAACGAGGCTAACG |
| 39 | CDH1_Rev | TTCACATCCAGCACATCC |
| 40 | CDH2_Fwd | ATGTGCCGGATAGCGGGAGC |
| 41 | SNAI1_Fwd | GCCTAGCGAGTGGTTCTTCT |
| 42 | SNAI1_Rev | TAGGGCTGCTGGAAGGTAAA |
| 43 | VIM_Fwd | GCCTCAGATTCAGGAACAGC |
| 44 | VIM_Rev | GCTTCAACGGCAAGTTCTC |
