# Supplementary material for: PRMT5 regulates alternative splicing of TCF3 under hypoxia to promote EMT and invasion in breast cancer
Source: PLoS Biol. 2025 Oct 28;23(10):e3003444. doi: 10.1371/journal.pbio.3003444 (PMC12585103; doi:10.1371/journal.pbio.3003444)
Supplement: S2 Table — (DOCX) [file pbio.3003444.s002.docx]

**S2 Table: List of shRNA sequence**

| S  No  . | Gene | Sequence |
| --- | --- | --- |
| 1 | sh*PRMT5*_1 | CCGGAGGGACTGGAATACGCTAATTCTCGAGAATTAGCGTA TTCCAGTCCCTTTTTTG |
| 2 | sh*PRMT5*_2 | CCGGGCGTTTCAAGAGGGAGTTCATCTCGAGATGAACTCCC TCTTGAAACGCTTTTTG |
| 3 | sh*CTCF*_1 | CCGGGCGGAAAGTGAACCCATGATACTCGAGTATCATGGGT TCACTTTCCGCTTTTT |
| 4 | sh*CTCF*_2 | CCGGGCTGTGTTTCATGAGCGCTATCTCGAGATAGCGCTCAT GAAACACAGCTTTTT |
| 5 | sh*MECP2*_1 | CCGGCTGGGAAGTATGATGTGTATTCTCGAGAATACACATC ATACTTCCCAGTTTTT |
| 6 | sh*MECP2*_2 | CCGGCGTCTGCAAAGAGGAGAAGATCTCGAGATCTTCTCCT CTTTGCAGACGTTTTT |
| 7 | sh*PTBP1*_1 | CCGGCGTCGTCAAAGGATTCAAGTTCTCGAGAACTTGAATC CTTTGACGACGTTTTT |
| 8 | sh*PTBP1*_2 | CCGGCCAGCCCATCTACATCCAGTTCTCGAGAACTGGATGT AGATGGGCTGGTTTTT |
| 9 | shCTRL | CCGGCGTGATCTTCACCGACAAGATCTCGAGATCTTGTCGG TGAAGATCACGTTTTT |
