# Supplementary material for: PRMT5 regulates alternative splicing of TCF3 under hypoxia to promote EMT and invasion in breast cancer
Source: PLoS Biol. 2025 Oct 28;23(10):e3003444. doi: 10.1371/journal.pbio.3003444 (PMC12585103; doi:10.1371/journal.pbio.3003444)
Supplement: S4 Table — (DOCX) [file pbio.3003444.s004.docx]

**S4 Table: List of antibodies**

| S No. | Name | Species | Make | Experiment/Dilutions | Catalogue |
| --- | --- | --- | --- | --- | --- |
| 1 | PRMT5 | Rabbit | Abcam | WB (1:1000) | ab151321 |
| 2 | CA9 | Rabbit | Abcam | IHC-F (1:100) | ab184006 |
| 3 | CTCF | Rabbit | CST | WB (1:1000) | #3418 |
| 4 | β-ACTIN | Rabbit | CST | WB (1:1000) | #8457s |
| 5 | VIMENTIN | Rabbit | Abcam | WB (1:1000) | ab137321 |
| 6 | SNAIL | Rabbit | Proteintech | WB (1:1000) | 13099-1-AP |
| 7 | KERATIN 8/18 | Mouse | CST | WB (1:1000) | #4546 |
| 8 | Anti-Symmetric  Dimethyl-Histone H4 (Arg3) | Rabbit | PTM biolabs | ChIP (1:100)  WB (1:1000) | PTM-639 |
| 9 | Anti-Symmetric  Dimethyl-Histone H3 (Arg8) | Rabbit | PTM biolabs | ChIP (1:100)  WB (1:1000) | PTM-672 |
| 10 | MeCP2 | Rabbit | CST | ChIP (1:100)  WB (1:1000) | #3456 |
| 11 | 5-Methyl Cytosine | Rabbit | CST | MeDIP (1:200) | #28692s |
| 12 | E-CAD | Rabbit | Abcam | WB (1:3000) | ab40772 |
| 13 | Rpb1 CTD | Mouse | CST | ChIP (1:200) | #2629 |
| 14 | PTBP1 | Rabbit | CST | PAR-CLIP (1:200)  WB (1:1000) | #72669 |
| 15 | DNMT3A | Rabbit | CST | ChIP (1:50)  WB (1:500) | #3598 |
| 17 | E2A | Rabbit | CST | WB (1:1000) | #12258 |
| 18 | Normal Rabbit IgG | Rabbit | CST | ChIP/MeDIP/PAR-CLIP (1:20) | #2729 |
| 179 | Normal Mouse IgG | Mouse | Millipore | ChIP (1:50) | NI03 |
| 18 | Anti-Flag tag | Rat | Novus  Biologicals | WB (1:1000) | NBP1-06712SS |
