# Supplementary material for: PRMT5 regulates alternative splicing of TCF3 under hypoxia to promote EMT and invasion in breast cancer
Source: PLoS Biol. 2025 Oct 28;23(10):e3003444. doi: 10.1371/journal.pbio.3003444 (PMC12585103; doi:10.1371/journal.pbio.3003444)

RAW BLOTS

SourceData1E

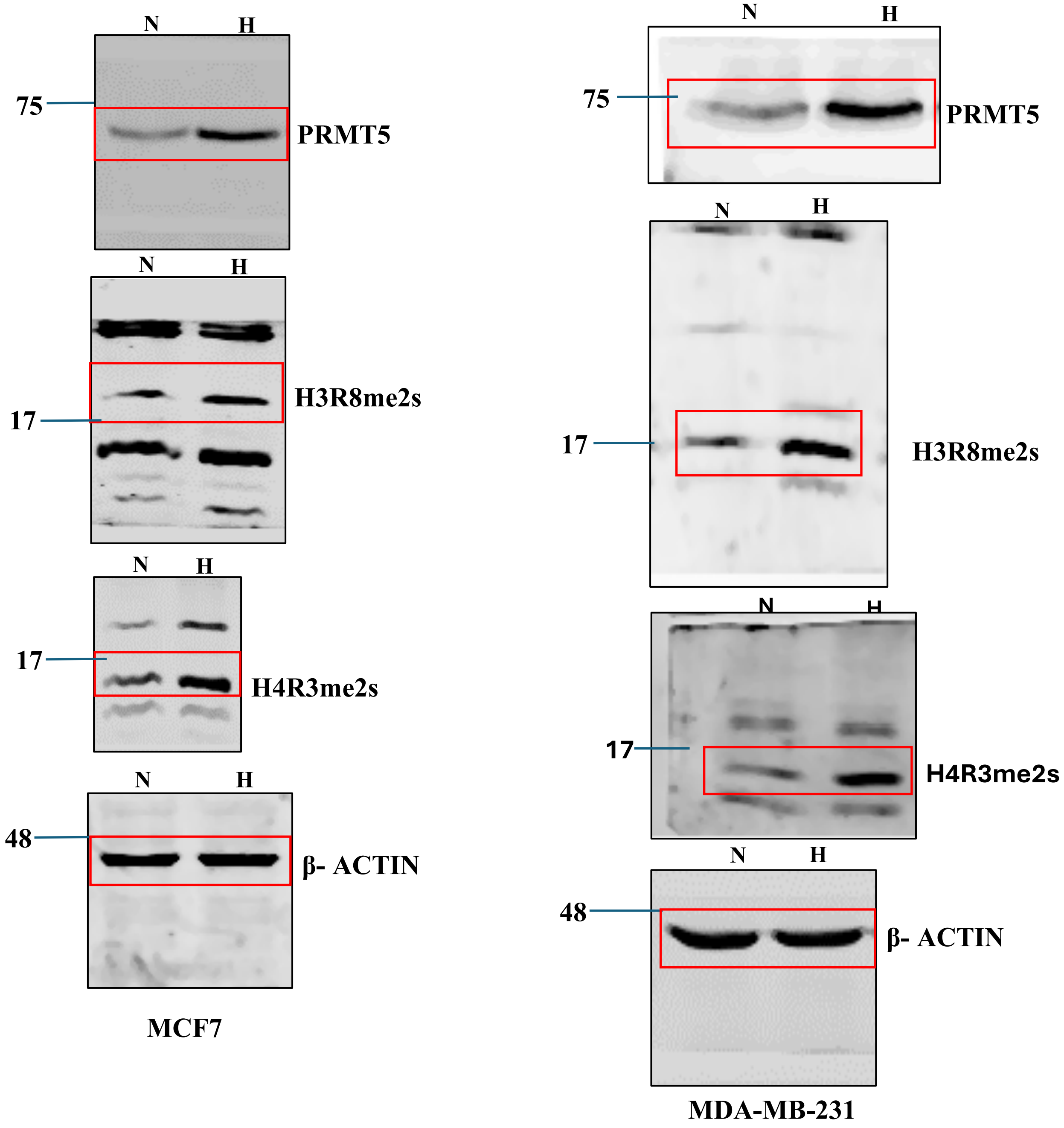

SourceData1G

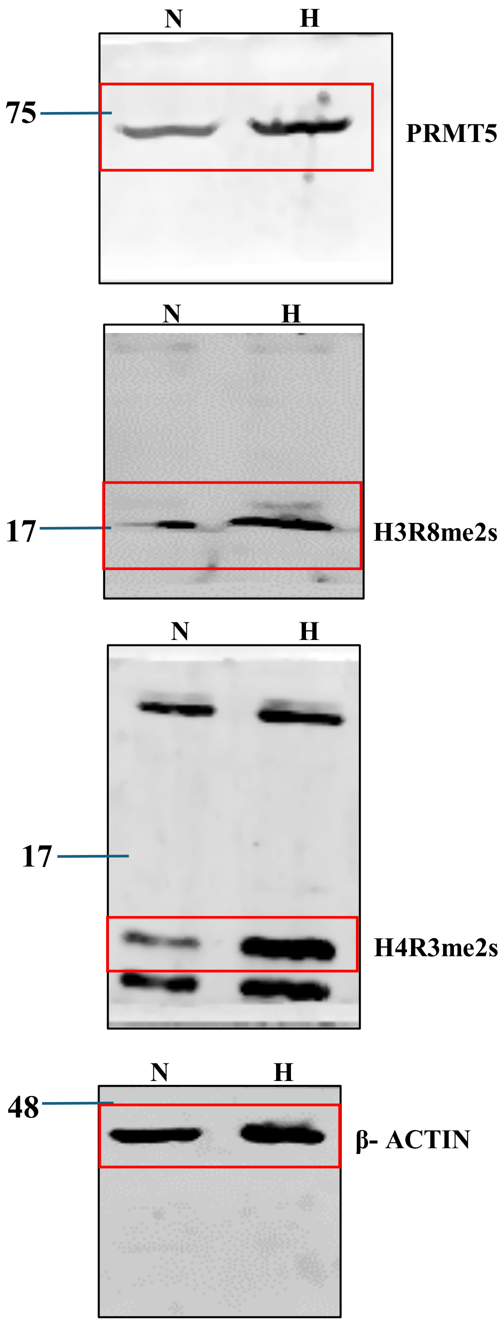

BC8322

SourceData2D

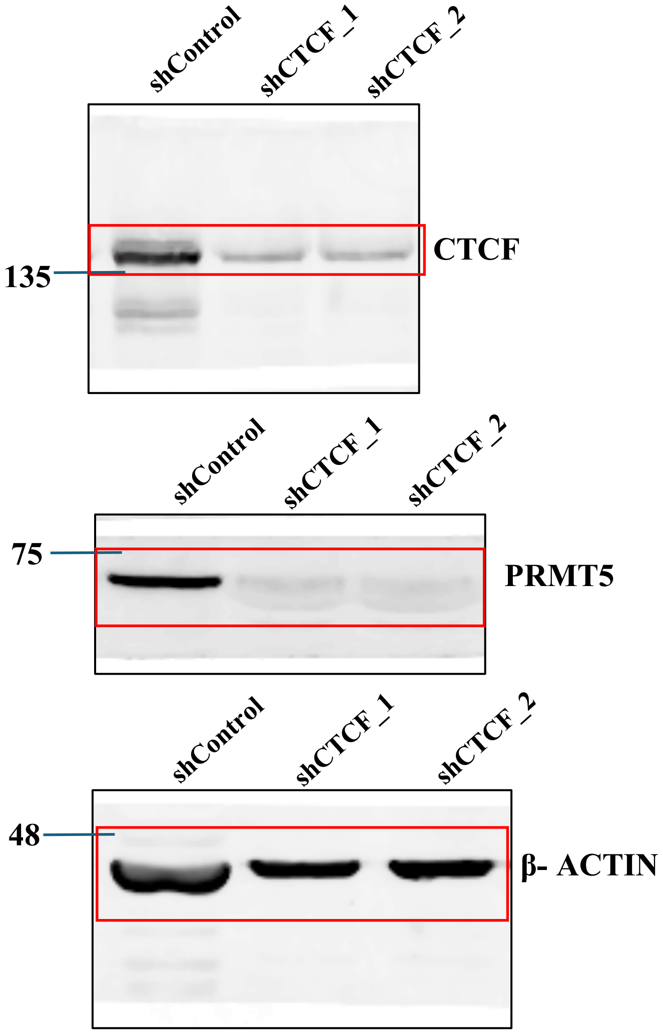

MCF7

SourceData2K

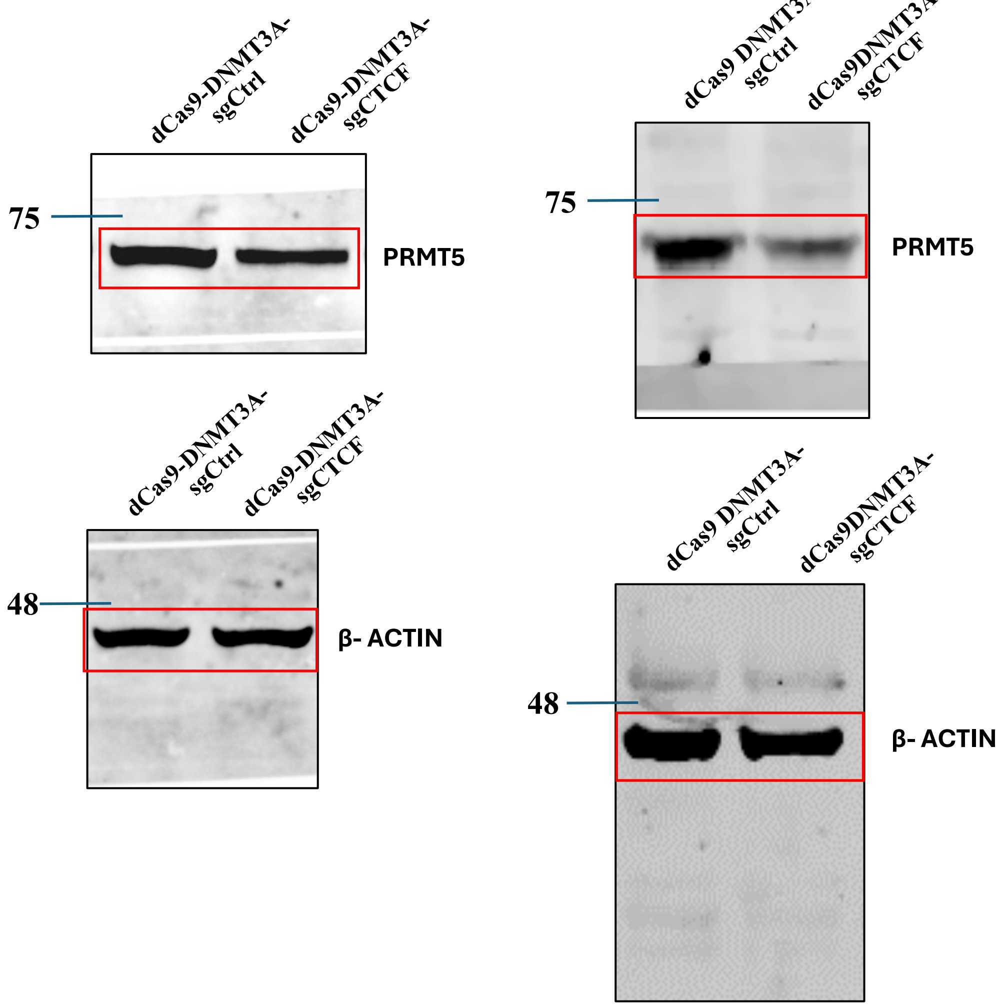

SourceDataS2D

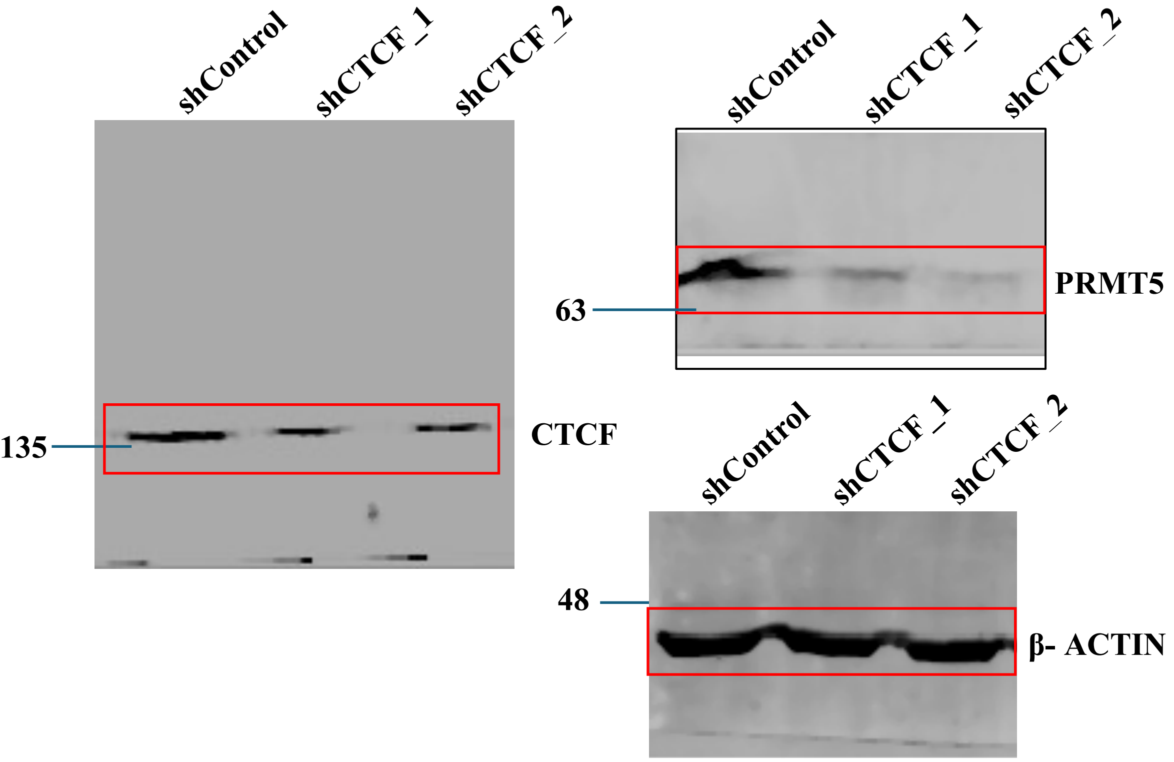

SourceData3B

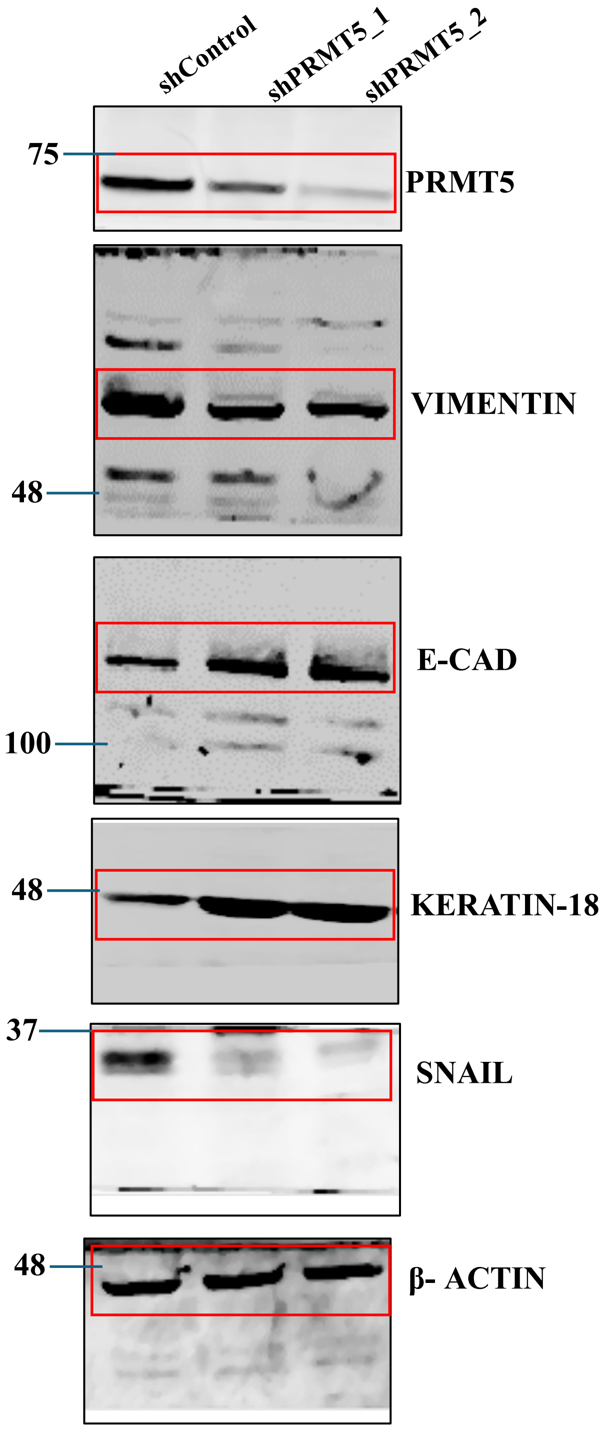

SourceData3C

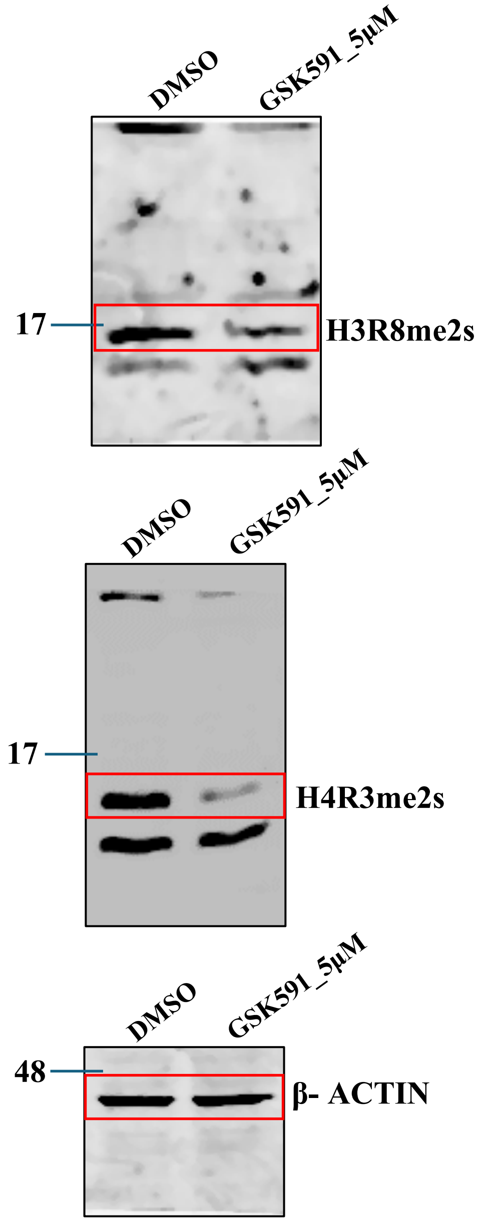

SourceData3E

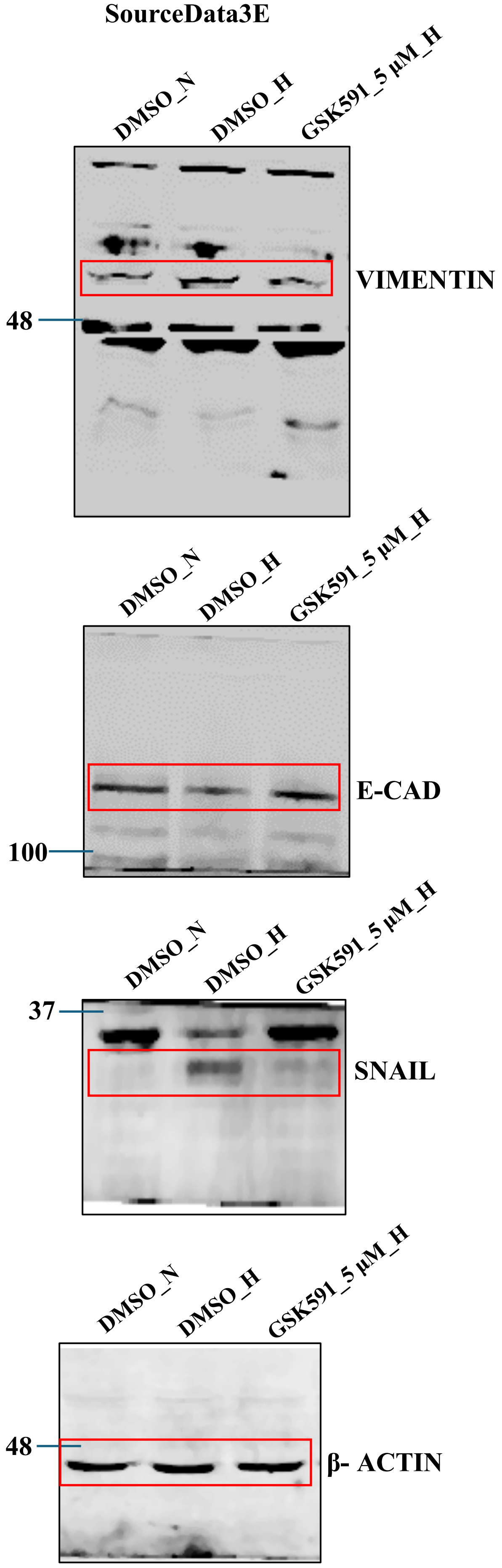

SourceDataS3B

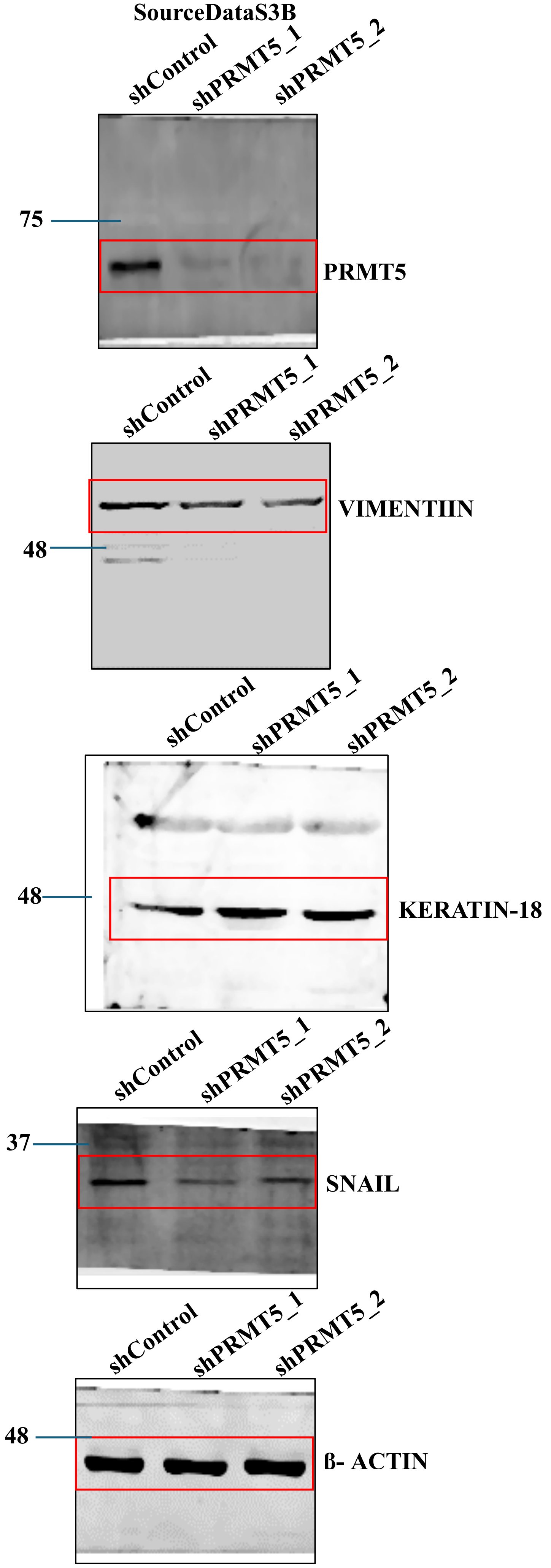

SourceDataS3C

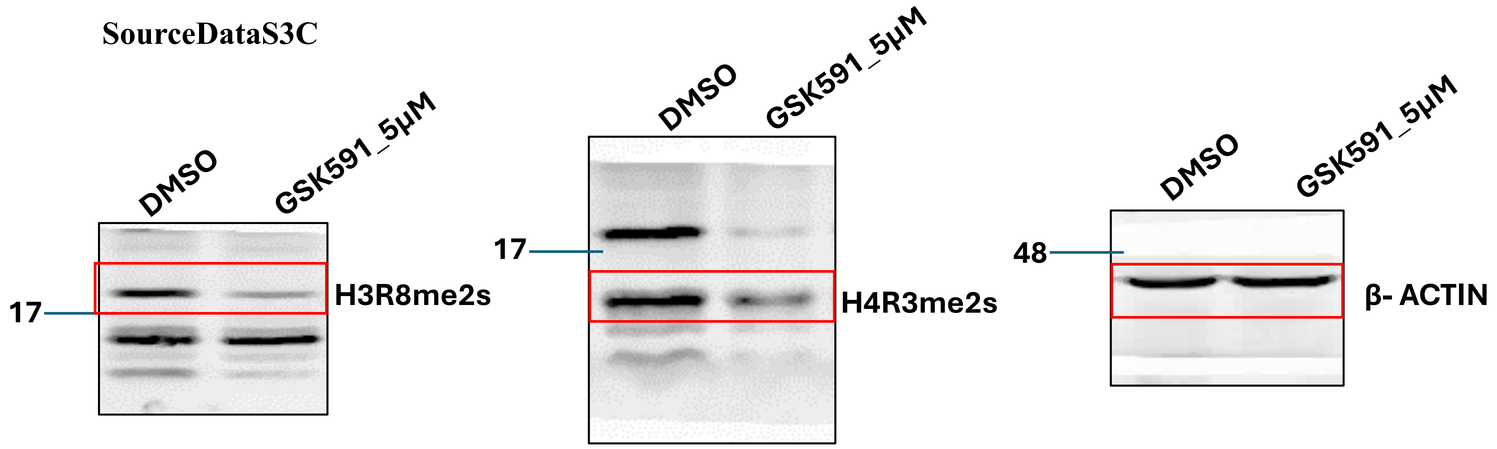

SourceDataS3F

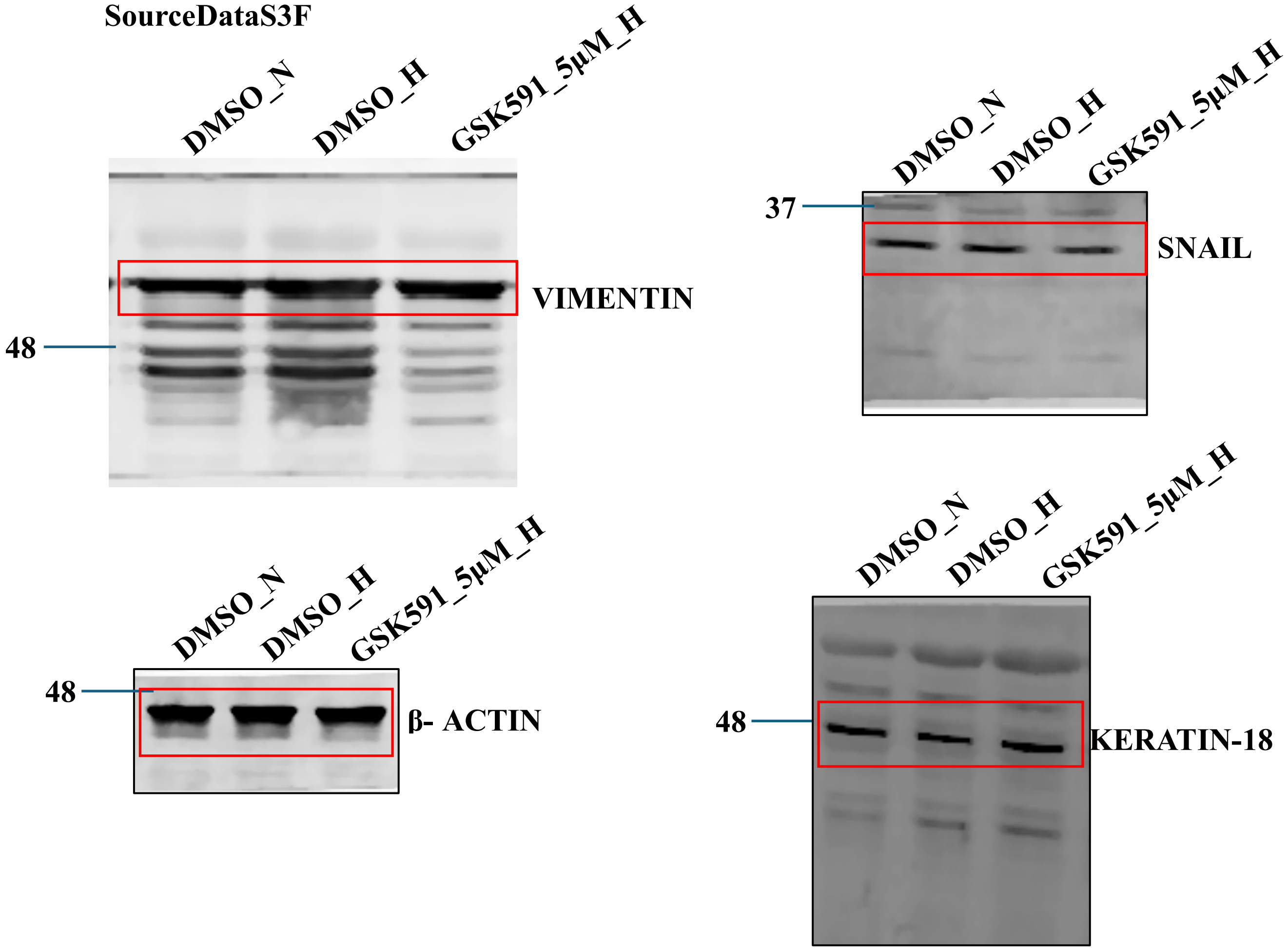

SourceData4G

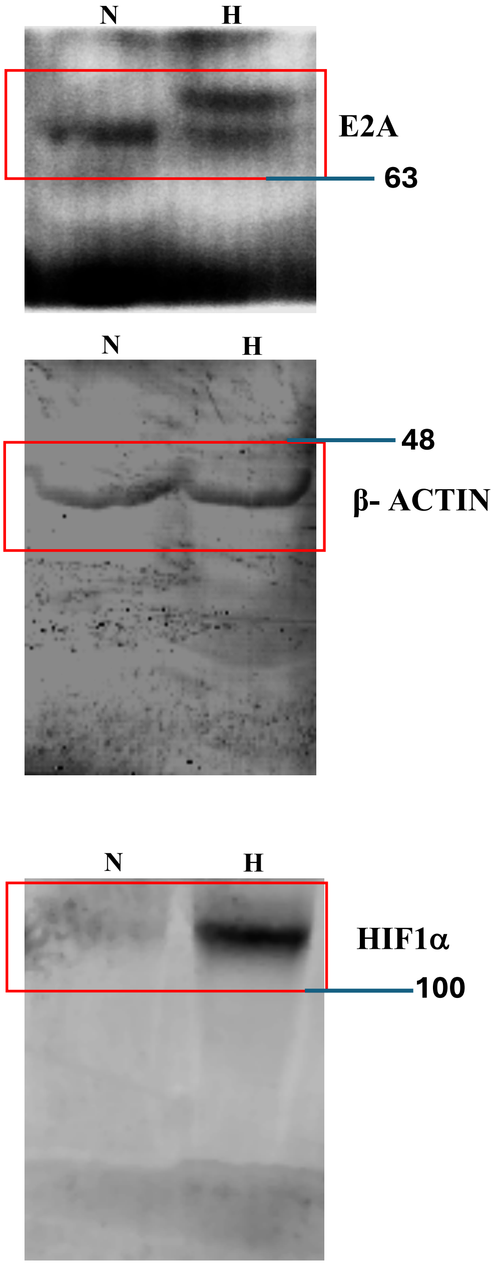

SourceDataS4B

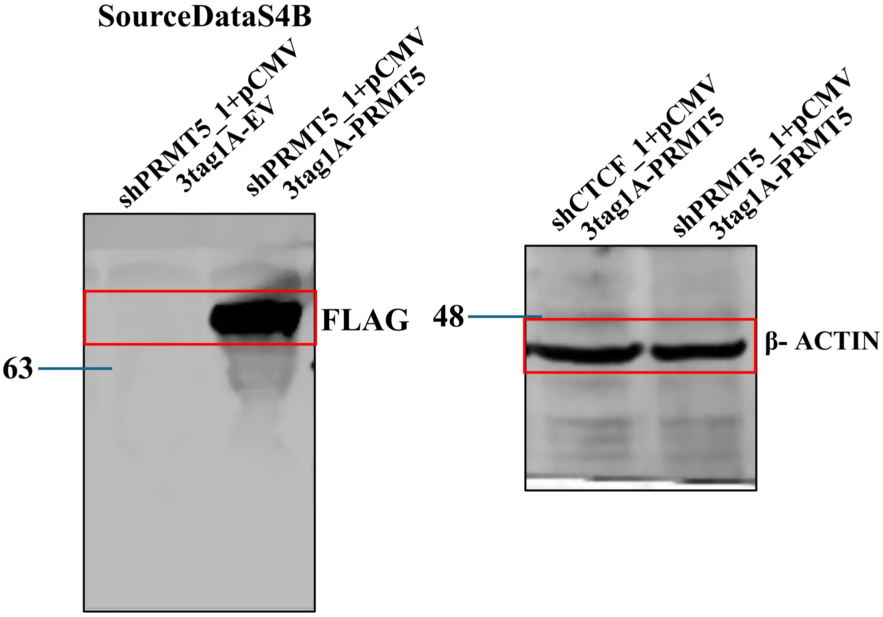

SourceDataS4D

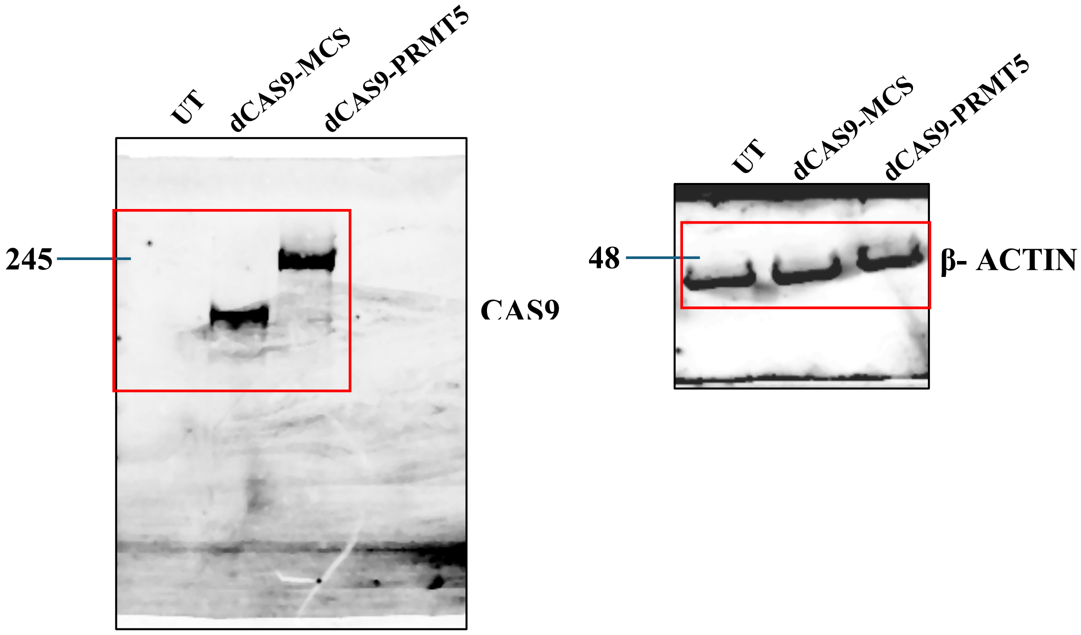

SourceDataS4K

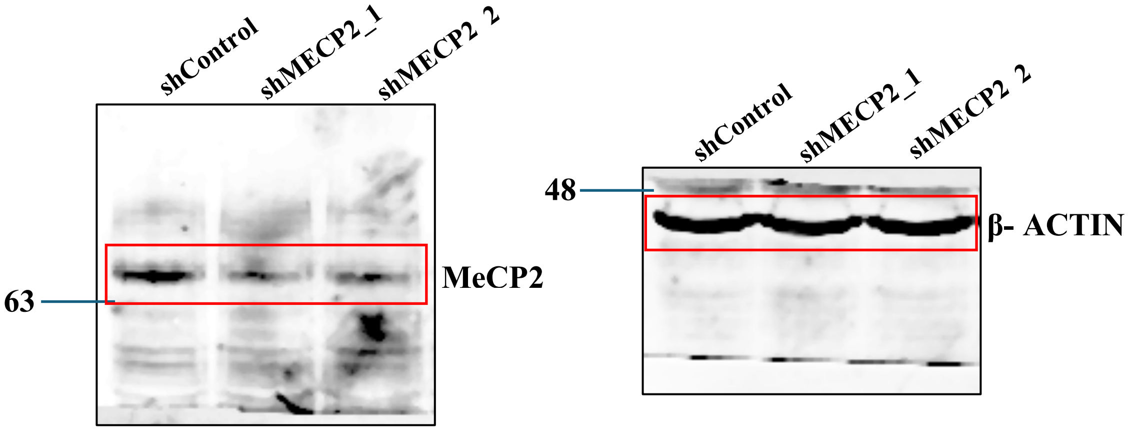

SourceDataS5C

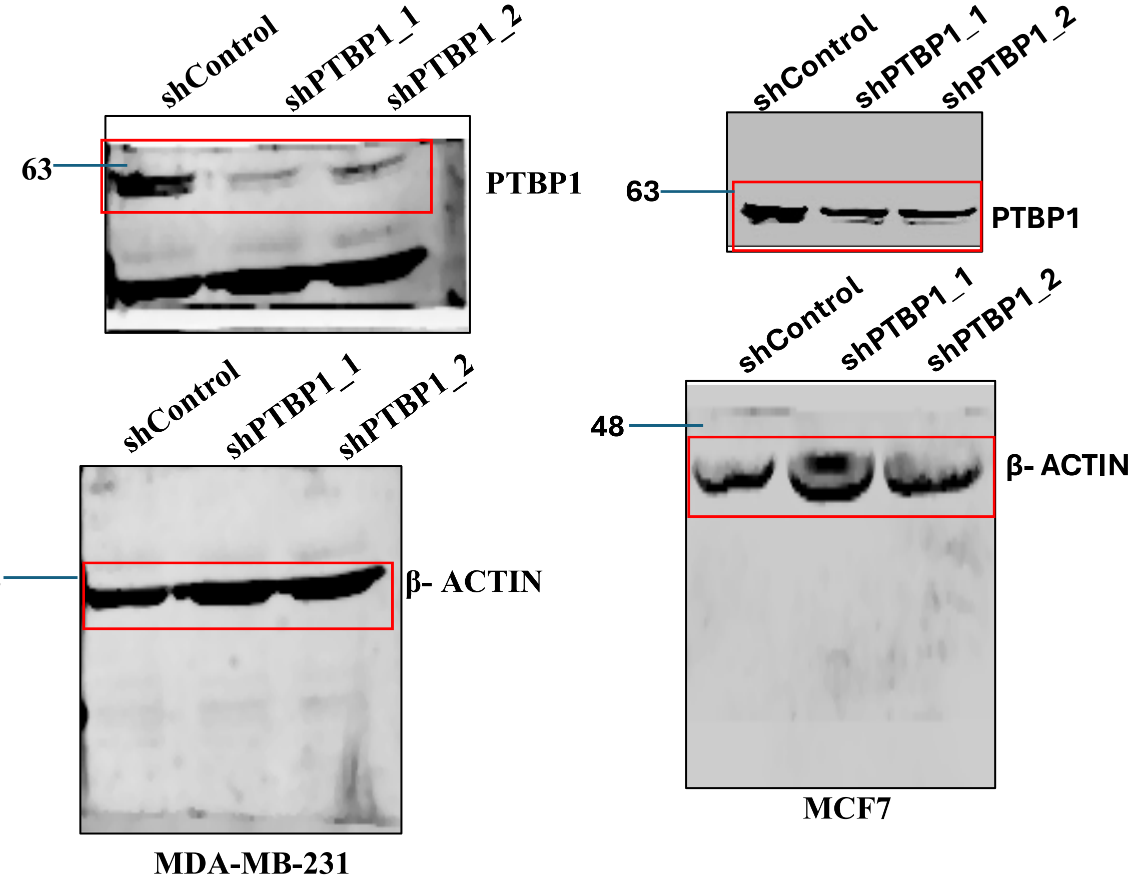

SourceDataS5D

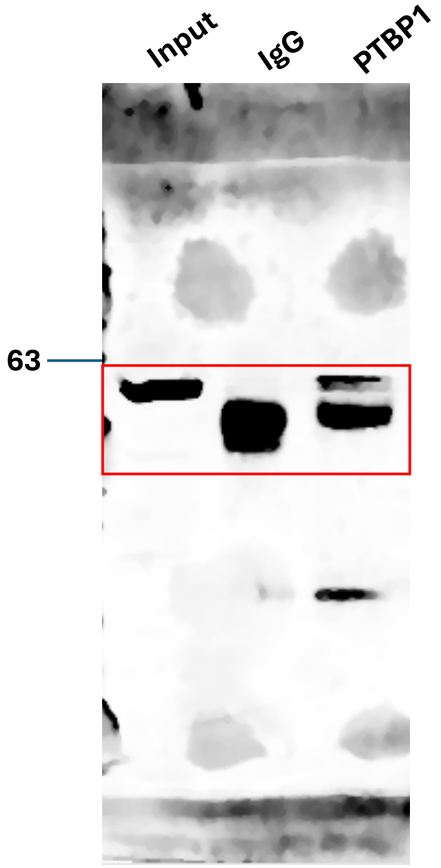

SourceDataS6A

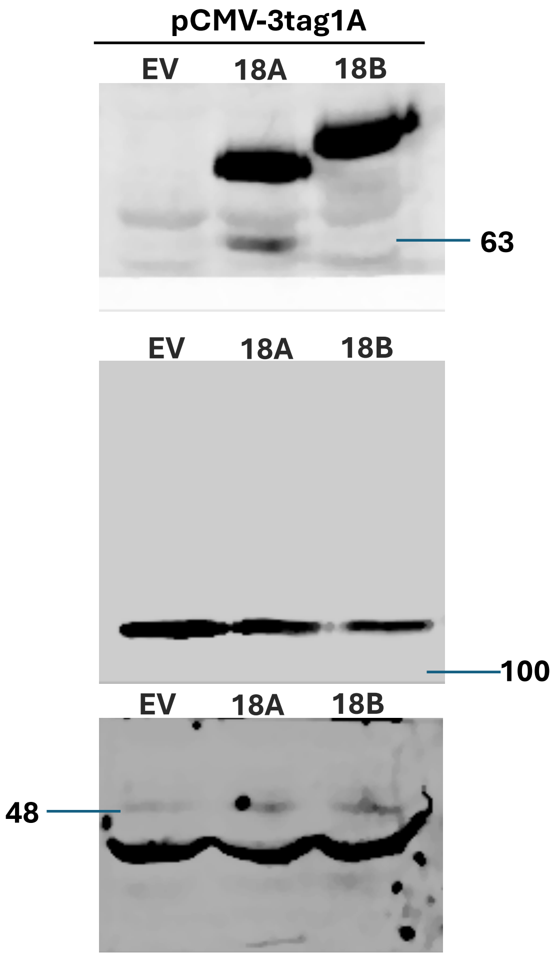

SourceData6B

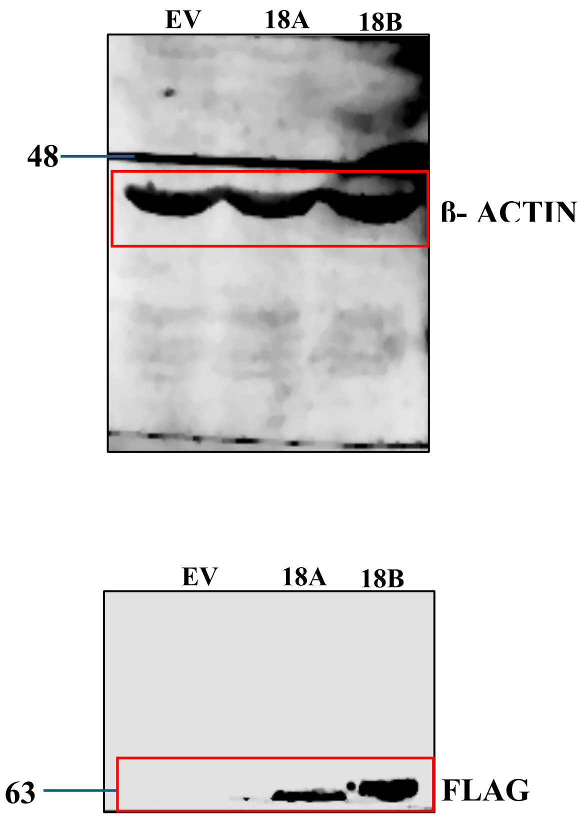

SourceData 6H

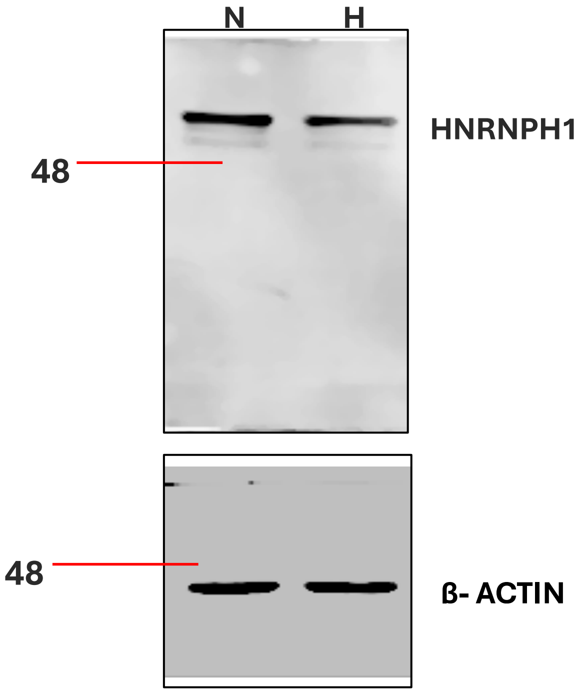

Supplement: S1 Raw Images — (PDF) [file pbio.3003444.s005.pdf]
