# Supplementary material for: PRMT5 regulates alternative splicing of TCF3 under hypoxia to promote EMT and invasion in breast cancer
Source: PLoS Biol. 2025 Oct 28;23(10):e3003444. doi: 10.1371/journal.pbio.3003444 (PMC12585103; doi:10.1371/journal.pbio.3003444)
Supplement: S1 Fig — A) Immunohistochemistry-Fluorescence (IHC-F) analysis showing PRMT5 and CA9 expression in tumor sections of breast cancer patients. Scale bar 20 µm. (DOCX) [file pbio.3003444.s007.docx]

**Supplementary Figure 1.**

**
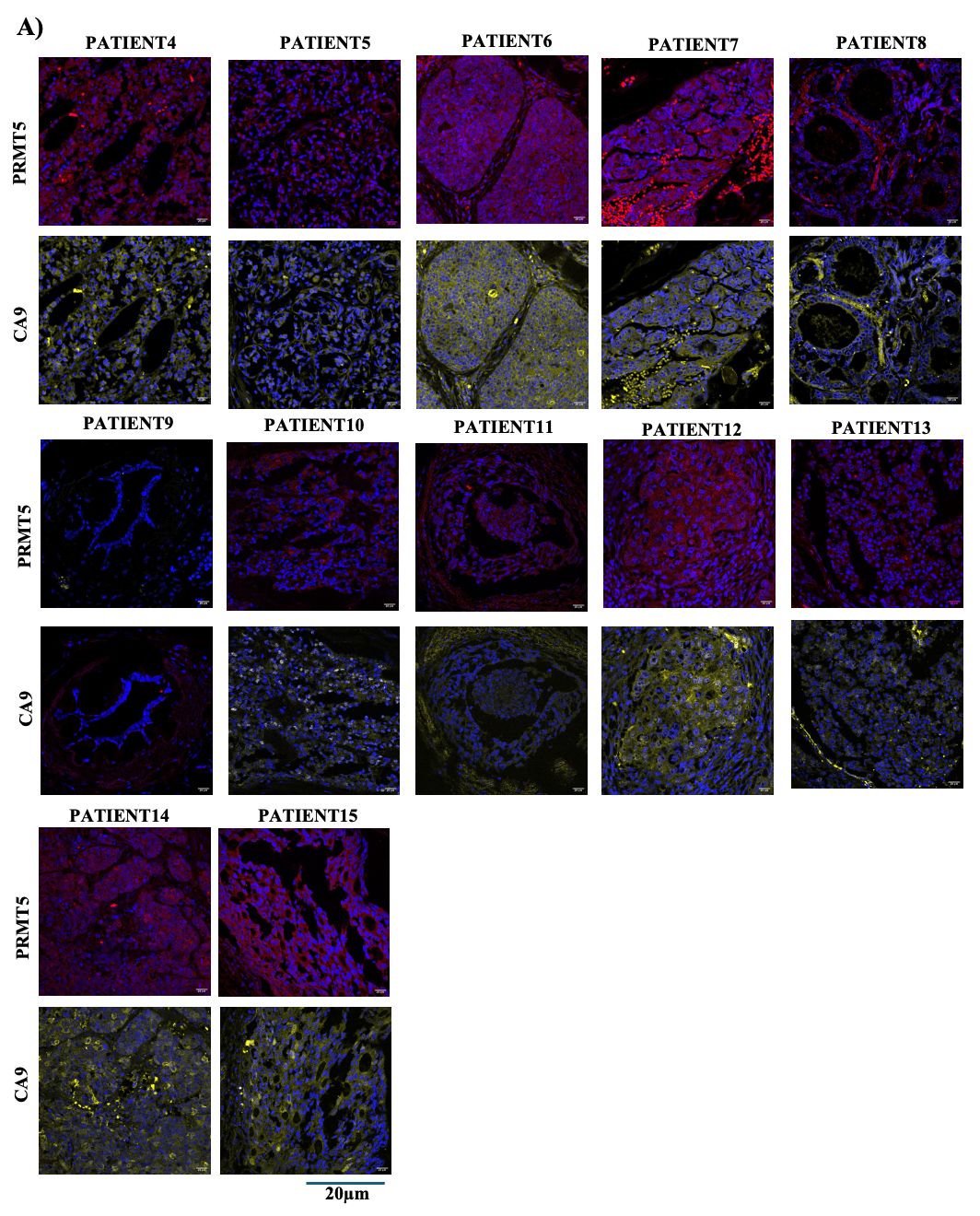
**

**S1 Figure. PRMT5 is upregulated under hypoxia in clinical breast cancer patient samples**

A) Immunohistochemistry-Fluorescence (IHC-F) analysis showing PRMT5 and CA9 expression in tumor sections of breast cancer patients. Scale bar 20µm.
