# Supplementary material for: PRMT5 regulates alternative splicing of TCF3 under hypoxia to promote EMT and invasion in breast cancer
Source: PLoS Biol. 2025 Oct 28;23(10):e3003444. doi: 10.1371/journal.pbio.3003444 (PMC12585103; doi:10.1371/journal.pbio.3003444)
Supplement: S2 Fig — A) Luciferase assay showing an increase in luciferase activity under hypoxia in MCF7 cells. B) Immunoblot showing decrease in PRMT5 expression upon CTCF KD under hypoxia in MDA-MB-231 cells. C) Transcription factors binding sites obtained from ChIP-Atlas showing presence of CTCF binding site at PRMT5 promoter in breast cells. D) MeDIP-qPCR showing decrease in DNA methylation at PRMT5 promoter in MDA-MB-231 cells normoxia versus hypoxia. E) CTCF Chip qPCR showing enrichment in CTCF binding at PRMT5 promoter in MDA-MB-231 cells normoxia versus hypoxia. F) Chromatogram showing mutations induced in the PRMT5 promoter luciferase (−1,500) construct. G) Luciferase assay showing decrease in luciferase activity in PRMT5 luciferase promoter construct (−1,500) harboring a mutated CTCF binding site. Error bars, mean ± SEM; two-tailed t test, one-way ANOVA. *p < 0.05, **p < 0.01, ***p < 0.001, ****p < 0.0001, n = 3 biological replicates. Numerical data of (A), (D–E), (G) available in S1 Data, sheet “Figure S2.” (DOCX) [file pbio.3003444.s008.docx]

**Supplementary Figure 2.**


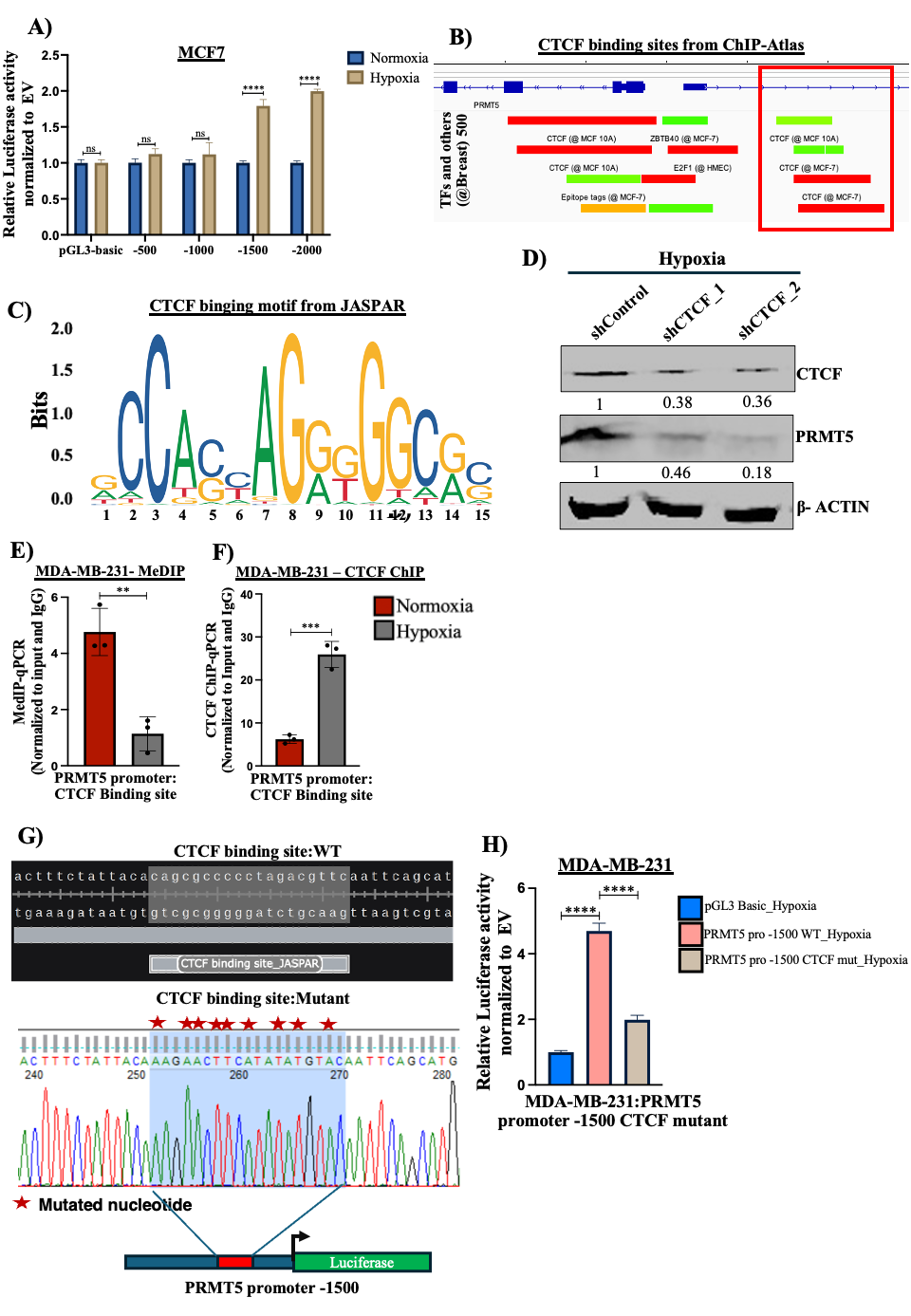


**S2 Figure. CTCF is responsible for upregulation of PRMT5 under hypoxia in breast cancer cells**

A) Luciferase assay showing an increase in luciferase activity under hypoxia in MCF7 cells. B) Immunoblot showing decrease in PRMT5 expression upon CTCF KD under hypoxia in MDA-MB-231 cells. C) Transcription factors binding sites obtained from ChIP-Atlas showing presence of CTCF binding site at PRMT5 promoter in breast cells. D) MeDIP-qPCR showing decrease in DNA methylation at PRMT5 promoter in MDA-MB-231 cells normoxia vs hypoxia. E) CTCF Chip qPCR showing enrichment in CTCF binding at PRMT5 promoter in MDA-MB-231 cells normoxia vs hypoxia. F) Chromatogram showing mutations induced in the PRMT5 promoter luciferase (-1500) construct. G) Luciferase assay showing decrease in luciferase activity in PRMT5 luciferase promoter construct (-1500) harboring a mutated CTCF binding site. Error bars, mean ± SEM; two-tailed t test, one way ANOVA.
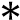
 *p* < 0.05,
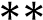
 *p* < 0.01,
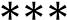
 *p* < 0.001,
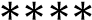
 *p* < 0.0001, n = 3 biological replicates. Numerical data of (A), (D-E), (G) available in S1_Data.xlsx, sheet Figure S2.
