# Supplementary material for: PRMT5 regulates alternative splicing of TCF3 under hypoxia to promote EMT and invasion in breast cancer
Source: PLoS Biol. 2025 Oct 28;23(10):e3003444. doi: 10.1371/journal.pbio.3003444 (PMC12585103; doi:10.1371/journal.pbio.3003444)
Supplement: S3 Fig — A) qRT-PCR depicting mRNA expression of EMT markers in shCTRL versus shPRMT5 MDA-MB-231 cells under hypoxia. B) Immunoblot showing protein level of EMT markers in shCTRL versus shPRMT5 MDA-MB-231 cells under hypoxia. C) Immunoblot showing decrease in histone marks HR38me2s and H4R3me2s upon vehicle (DMSO) versus 5 µM GSK591 treatment in MDA-MB-231 cells under hypoxia. D) qRT-PCR depicting mRNA expression of EMT markers upon vehicle (DMSO) versus 5 µM GSK591 treatment in MDA-MB-231 cells under hypoxia. E) Immunoblot showing protein level of EMT markers upon vehicle (DMSO) versus 5 µM GSK591 treatment in MDA-MB-231 cells under hypoxia. F, G) Matrigel invasion assay and its respective quantification in shControl versus shPRMT5 MDA-MB-231 cells under hypoxia. H) Matrigel invasion assay and its respective quantification (right side) upon vehicle (DMSO) versus 5 µM GSK591 treatment in MDA-MB-231 cells under hypoxia. Scale bar 2 µm. Error bars, mean ± SEM; two-tailed t test, one-way ANOVA. *p < 0.05, **p < 0.01, ***p < 0.001, ****p < 0.0001, n = 3 biological replicates. Numerical data of (A), (D) (G–H) available in S1 Data, sheet “Figure S3.” (DOCX) [file pbio.3003444.s009.docx]

**Supplementary Figure 4.**

**
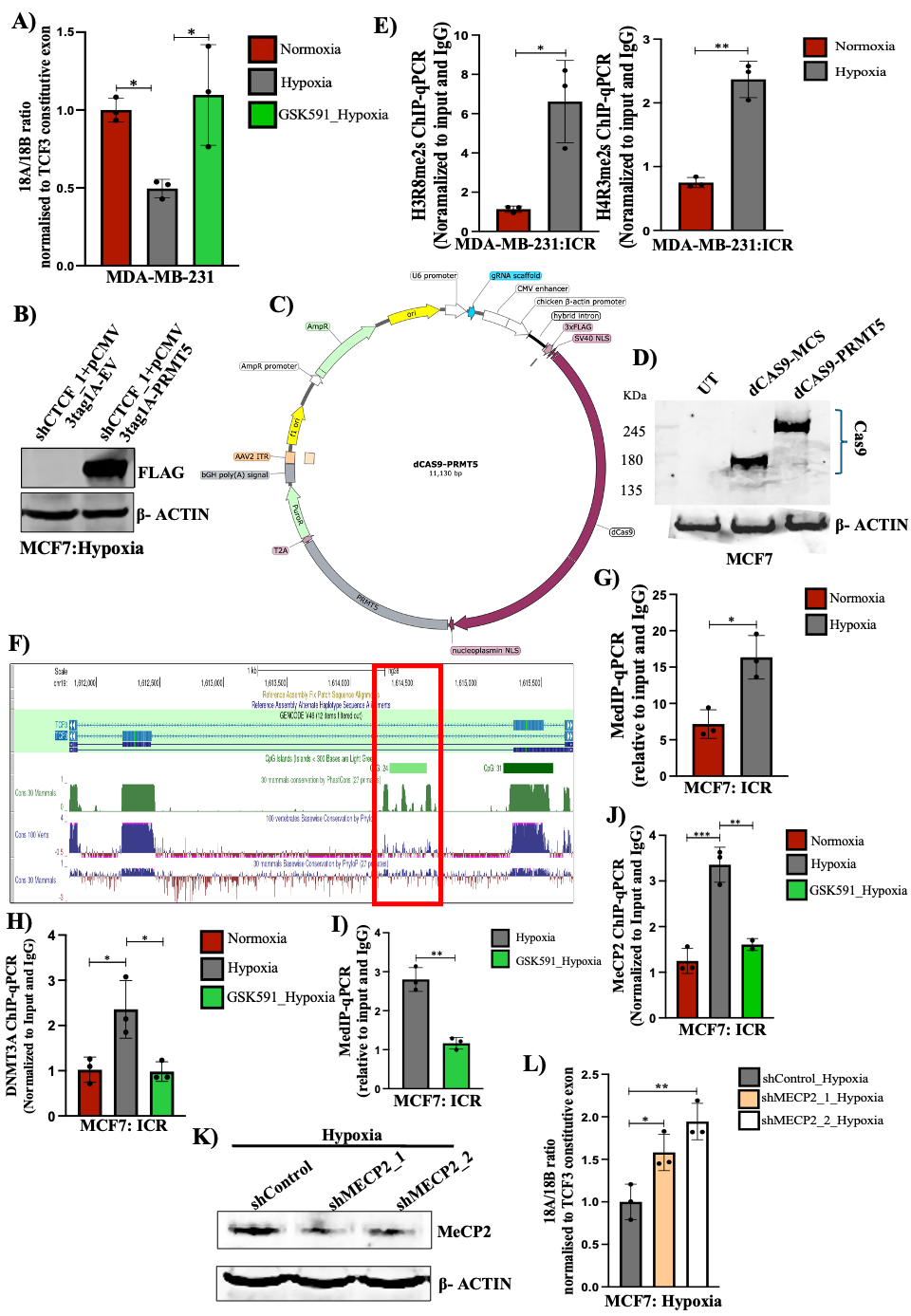
**

**S4 Figure. PRMT5 mediated histone methylation regulates *TCF3* alternative splicing under hypoxia**

A) qRT-PCR showing change in exon 18A/18B ratio upon vehicle (DMSO) vs 5µM GSK591 treatment in MDA-MB-231 cells. B) Immunoblot showing overexpression of PRMT5 in MCF7 cells under hypoxia. C) Vector map of dCAS9-PRMT5. D) Immunoblot showing Cas9 expression in MCF7 cells, untransfected control vs transfected with dCAS9-MCS or dCAS9-PRMT5 construct. E) H3R8me2s and H4R3me2s ChIP qPCR depicting increased histone symmetric arginine dimethylation at TCF3-ICR region in MDA-MB-231 cells, normoxia vs hypoxia F) Genome track from UCSC genome browser showing the presence of a CpG island at the TCF3-ICR region. G) MeDIP qPCR showing increase in DNA methylation at TCF3-ICR region in MCF7 cells, normoxia vs hypoxia. H) DNMT3A ChIP qPCR showing change in DNMT3A binding at TCF3-ICR region in MCF7 cells treated with DMSO (Nx vs Hx) or 5µM GSK591(Hx). I) MeDIP qPCR showing decrease in DNA methylation at TCF3-ICR region in MCF7 cells after treatment with DMSO vs 5µM GSK591under hypoxia. J) MeCP2 ChIP qPCR depicting change in MeCP2 binding at TCF3-ICR region in MCF7 cells treated with DMSO (Nx vs Hx) or 5µM GSK591(Hx). K) Immunoblot showing decrease in MeCP2 protein levels in shMECP2 MCF7 cells subjected to hypoxia. L) qRT-PCR showing increase in exon 18A/18B ratio in shMECP2 MCF7 cells under hypoxia. Error bars, mean ± SEM; two-tailed t test, one way ANOVA.
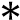
 *p* < 0.05,
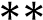
 *p* < 0.01,
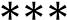
 *p* < 0.001,
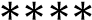
 *p* < 0.0001, n = 3 biological replicates. Numerical data of (A), (E), (G-J), (L) available in S1_Data.xlsx, sheet Figure S4.
