# Supplementary material for: PRMT5 regulates alternative splicing of TCF3 under hypoxia to promote EMT and invasion in breast cancer
Source: PLoS Biol. 2025 Oct 28;23(10):e3003444. doi: 10.1371/journal.pbio.3003444 (PMC12585103; doi:10.1371/journal.pbio.3003444)
Supplement: S5 Fig — A) RNA pol II ChIP qPCR demonstrating change in RNA pol II occupancy at TCF3-ICR region in MDA-MB-231 cells treated with DMSO (Nx versus Hx) or 5 µM GSK591(Hx). B) SpliceAid image showing binding of different splicing factors at the TCF3-ICR RNA. C) Immunoblot showing reduction in PTBP1 protein level upon knock down of PTBP1 in MCF7 and MDA-MB-231 cells under hypoxia. D) Immunoblot showing PTBP1 in PTBP1 pulldown upon PAR-CLIP. E) Sanger sequencing traces analyzed in DECODR showing CRISPR/Cas9 edited sequences. F) Plot from DECODR showing Indel distribution. G) Matrigel invasion assay and its respective quantification upon pCMV-3tag-1A- EV versus TCF3-18A versus TCF3-18B overexpression in MDA-MB-231 cells. Error bars, mean ± SEM; two-tailed t test, one-way ANOVA. *p < 0.05, **p < 0.01, ***p < 0.001, ****p < 0.0001, n = 3 biological replicates. Numerical data of (A) available in S1 Data, sheet “Figure S5.” (DOCX) [file pbio.3003444.s011.docx]

**Supplementary Figure 5.**

**
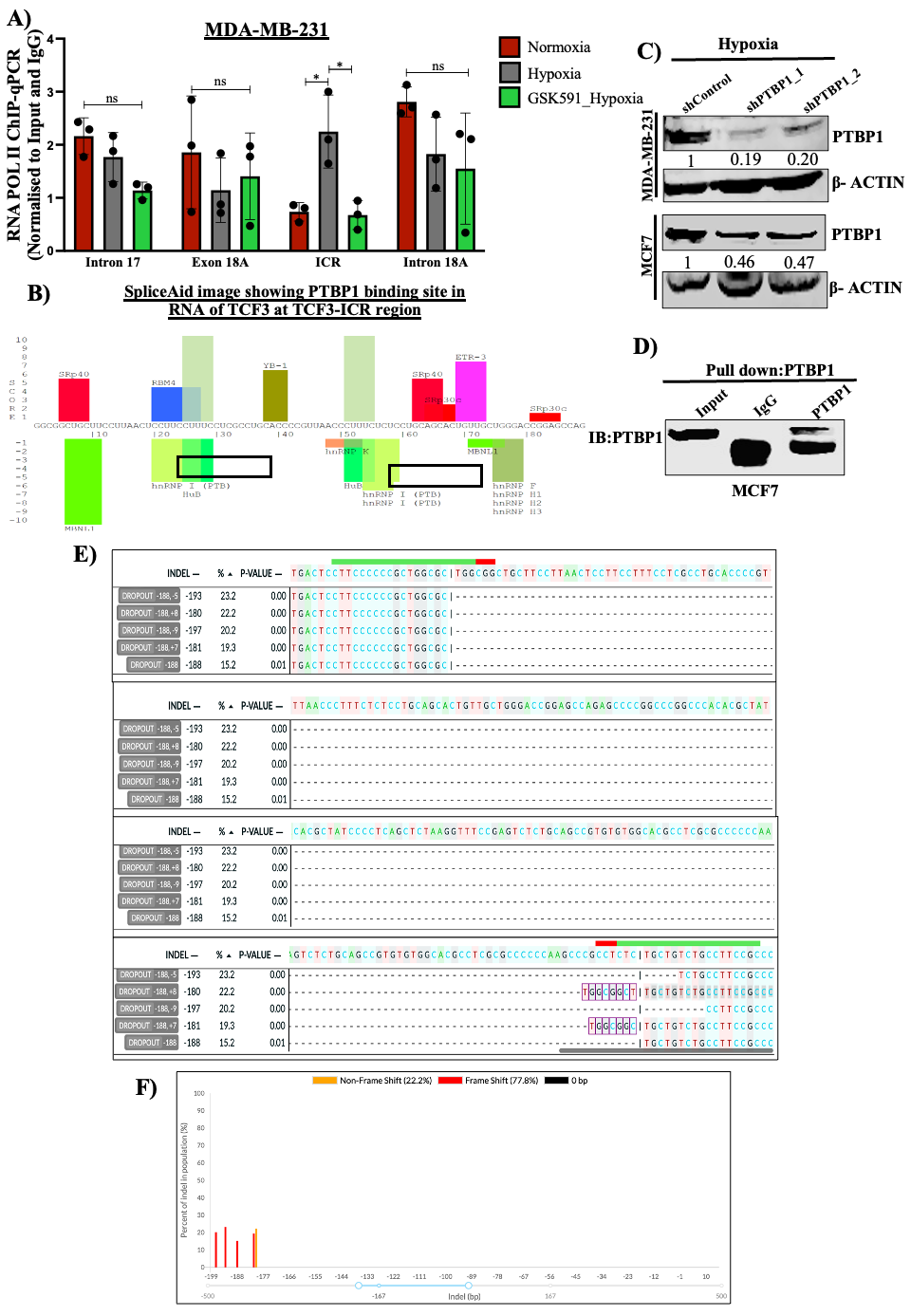
**

**S5 Figure**. **PRMT5 mediated changes at the ICR region is necessary for *TCF3* alternative splicing**

A) RNA pol II ChIP qPCR demonstrating change in RNA pol II occupancy at TCF3-ICR region in MDA-MB-231 cells treated with DMSO (Nx vs Hx) or 5µM GSK591(Hx). B) SpliceAid image showing binding of different splicing factors at the TCF3-ICR RNA. C) Immunoblot showing reduction in PTBP1 protein level upon knock down of PTBP1 in MCF7 and MDA-MB-231 cells under hypoxia. D) Immunoblot showing PTBP1 in PTBP1 pulldown upon PAR-CLIP. E) Sanger sequencing traces analyzed in DECODR showing CRISPR/Cas9 edited sequences. F) Plot from DECODR showing Indel distribution. G) Matrigel invasion assay and its respective quantification upon pCMV-3tag-1A- EV vs TCF3-18A vs TCF3-18B overexpression in MDA-MB-231 cells. Error bars, mean ± SEM; two-tailed t test, one way ANOVA.
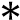
 *p* < 0.05,
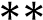
 *p* < 0.01,
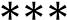
 *p* < 0.001,
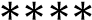
 *p* < 0.0001, n = 3 biological replicates. Numerical data of (A) available in S1_Data.xlsx, sheet Figure S5.
