# Supplementary material for: PRMT5 regulates alternative splicing of TCF3 under hypoxia to promote EMT and invasion in breast cancer
Source: PLoS Biol. 2025 Oct 28;23(10):e3003444. doi: 10.1371/journal.pbio.3003444 (PMC12585103; doi:10.1371/journal.pbio.3003444)
Supplement: S6 Fig — A) Immunoblot showing reduction in E-Cad expression upon TCF3-18B isoform overexpression in MCF7 cells. B) Immunoblot showing over expression of TCF3 18A or TCF3-18B isoform detected using anti FLAG antibody. C) Chromatogram depicting TCF3 exon-18A and exon-18B over expression construct cloned in pCMV-3tag1A vector. D) Matrigel invasion assay and its respective quantification upon pCMV-3tag-1A-EV versus TCF3-18A versus TCF3-18B overexpression in MDA-MB-231 and MCF7 cells. E) Matrigel invasion assay and its respective quantification in shPRMT5 MCF7 cells upon transfection of dCAS9-PRMT5-EV or dCAS9-PRMT5-sgICR under hypoxia. Scale bar, 2 µm. Error bars, mean ± SEM; two-tailed t test, one-way ANOVA. *p < 0.05, **p < 0.01, ***p < 0.001, ****p < 0.0001, n = 3 biological replicates. Numerical data of (D–E) available in S1 Data, sheet “Figure S6.” (DOCX) [file pbio.3003444.s012.docx]

**
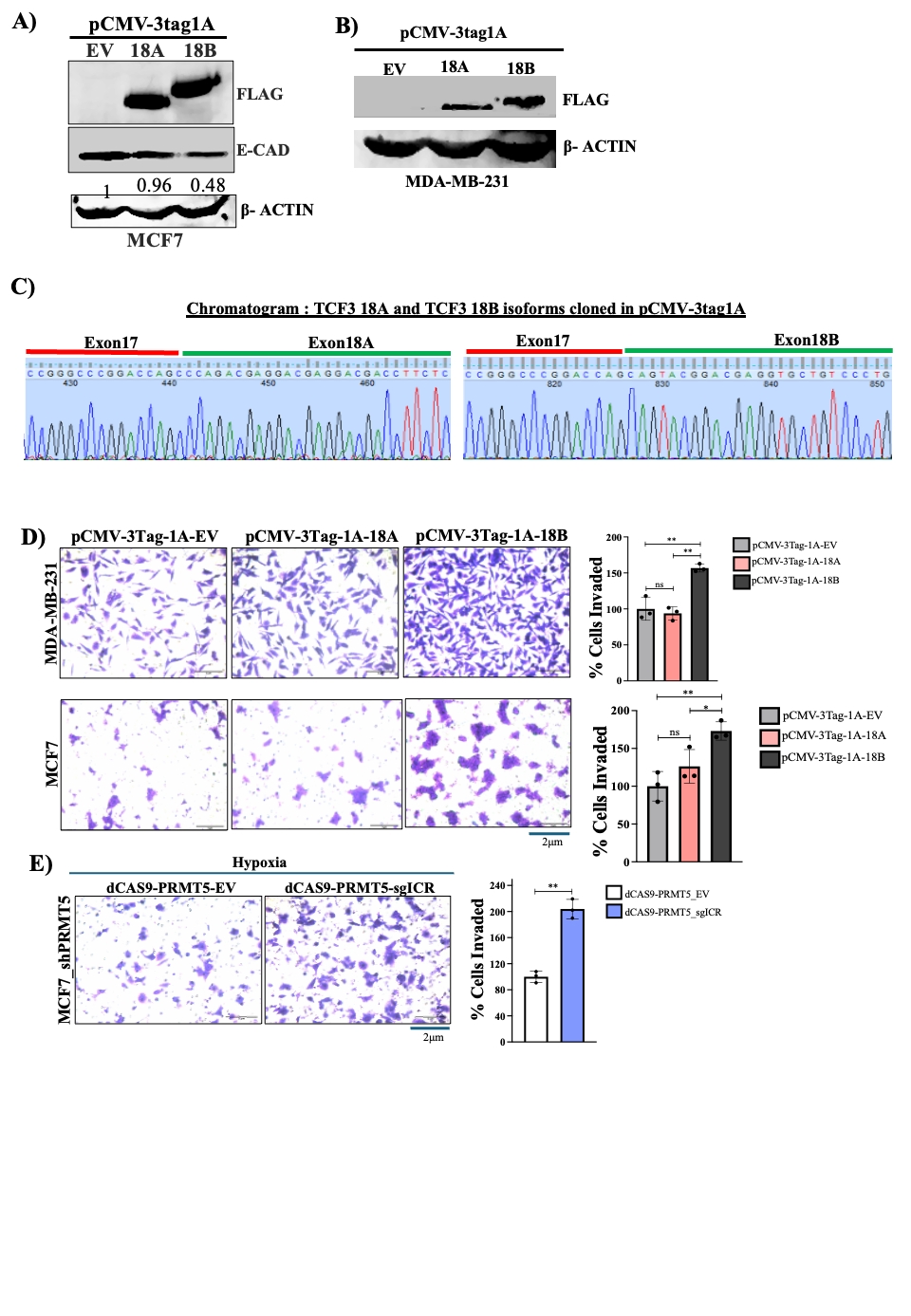
Supplementary Figure 6.**

**S6 Figure. TCF3-18B (E47) isoform promotes invasion of breast cancer cells.**

A) Immunoblot showing reduction in E-Cad expression upon TCF3-18B isoform overexpression in MCF7 cells. B) Immunoblot showing over expression of TCF3 18A or TCF3-18B isoform detected using anti FLAG antibody. C) Chromatogram depicting TCF3 exon-18A and exon-18B over expression construct cloned in pCMV-3tag1A vector. D) Matrigel invasion assay and its respective quantification upon pCMV-3tag-1A- EV vs TCF3-18A vs TCF3-18B overexpression in MDA-MB-231 and MCF7 cells. E) Matrigel invasion assay and its respective quantification in shPRMT5 MCF7 cells upon transfection of dCAS9-PRMT5-EV or dCAS9-PRMT5-sgICR under hypoxia. Scale bar, 2 µm. Error bars, mean ± SEM; two-tailed t test, one way ANOVA.
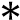
 *p* < 0.05,
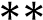
 *p* < 0.01,
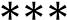
 *p* < 0.001,
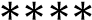
 *p* < 0.0001, n = 3 biological replicates. Numerical data of (D-E) available in S1_Data.xlsx, sheet Figure S6.
