# Supplementary material for: PRMT5 regulates alternative splicing of TCF3 under hypoxia to promote EMT and invasion in breast cancer
Source: PLoS Biol. 2025 Oct 28;23(10):e3003444. doi: 10.1371/journal.pbio.3003444 (PMC12585103; doi:10.1371/journal.pbio.3003444)
Supplement: Attachment — Submitted filename: Rebuttal_PLOS Biology.pdf [file pbio.3003444.s016.pdf]

Reviewer #1: In this paper, the authors propose that PRMT5 is upregulated in hypoxic conditions and that it controls the splicing of TCF3 increasing the expression of the E47 isoform that contains Exon 18B that promotes a more invasive phenotype. Mechanistically, they propose that under hypoxic conditions, CTCF binds to the PRMT5 promoter leading to increased transcription, PRMT5 increases the methylation of H4R3 and H3R8 in nucleosomes that are within a regulatory intronic region of TCF3 gene that enables the recruitment of DNMT3A and DNA methylation, the recruitment of the methyl-reader MeCP2 that stalls RNA PolII leading to recruitment of the splicing factor PTBP1.

This is in an interesting paper as the mechanisms by which PRMT5 regulates splicing independent of spliceosome assembly is largely unknown, and there is a large amount of data and some nice use of the dCAS9 system for loci-specific protein recruitment. However, I am not convinced that the data is fully supportive of the model they propose in Figure 7, particularly the link between histone symmetric dimethylation at the TCF3 intronic region and alteration in splicing. As such, in many places the results are over-interpretated. The authors repeatedly state that an effect inhibitor induced by the PRMT5 GSK591 is evidence that histone methylation is the mechanism which PRMT5 regulates splicing at the TCF3 locus, however this cannot be said given that all PRMT5 substrate methylation, including that of splicing factors, will be reduced. This is a major concern as it alters the interpretation of the data and the model.

Author Response: We thank the reviewer for the valuable feedback and kind appreciation for the work conducted in this study. We believe that this critical feedback has enhanced our understanding and representation of our work.
We are immensely thankful to the reviewer for the comments on Figure 7 and the lack of substantial evidence to support our overall hypothesis. Considering these critical inputs, in our revised manuscript, we have performed additional experiments which substantiate our previous findings. In addition to all the experiments performed using the PRMT5 inhibitor GSK591, we performed additional validatory experiments using our dCas9-PRMT5 system which is locus specific and a better tool system to highlight the importance of PRMT5 mediated histone methylation in regulation of TCF3 alternative splicing. In corroboration with our previous observations made using PRMT5 inhibitor GSK591, we observed that site specific introduction of histone methylation at the TCF3-ICR locus via the dCas9-PRMT5 system significantly affects the alternative splicing pattern of TCF3. These additional observations strongly indicate that under hypoxia, PRMT5 regulates TCF3 alternative splicing via its ability to symmetrically dimethylate histones.

Moreover, whilst the data in Figure 1 is convincing, the literature already supports a role for PRMT5 in hypoxia (PRMT5 promotes tumour growth and the methylation of ULK1 in hypoxic conditions (PMID: 35246531); hypoxic treatment of human lung cells increases PRMT1 and PRMT5 expression (PMID: 24121109), PRMT5 expression is upregulated in hypoxic endothelial cells (PMID: 35531958)), which was not discussed in the introduction.
Author Response: We thank the reviewer for highlighting these missing references in the introduction section. PMID 35246531 paper shows that there is no difference in PRMT5 expression under hypoxia in LN229, Huh7 and HOK cell lines. PMID: 24121109 shows increase in PRMT5 expression under ischemia induced hypoxia in A549 cells which is not a breast cancer model. PMID: 35531958 shows increase in PRMT5 expression upon $CoCl_2$ induction in HUVEC cells under the context of ischemia induced hypoxia. These references have now been included in the introduction to highlight the importance of the role of PRMT5 in hypoxia to give the readers a sense of relevance in studying PRMT5 under hypoxia. Also, we would like to highlight that the regulation of PRMT5 expression was not previously reported. Additionally, the downstream effects of PRMT5 in the context of regulation of

alternative splicing via its ability to methylate histones were not explored under hypoxia in breast cancer models previously.

This paper has been hard to make comments due to a lack of page and line numbers. Please can this be included in revision.
Author Response: We thank the reviewer for pointing out the lack of line and page numbers. We have now made sure that page and line numbers are included in the revised version of the manuscript.

Major corrections:
1. Data in 1A that concludes that PRMT5 is the main PRMT whose expression is upregulated in hypoxia is not convincing, possibly because of a change of scale used between the normoxic and hypoxic sections. As such, it unclear how data in 1B is generated.
Author Response: We thank the reviewer for pointing out this discrepancy. We have now modified the heat map in a way that is easy to interpret (Rebuttal Fig 1A-B). The data in Fig 1B is a snippet of the data in Fig 1A pertaining to the expression of PRMT5 alone in Hypoxia high vs Hypoxia low stratified tumor patient microarray data. The breast cancer patient microarray data from GSE76250 was downloaded from https://xenabrowser.net/. These samples were then stratified as either hypoxia high or hypoxia low based on signature gene expression obtained from Hypoxia Hallmark signature from MSigDB. Samples with higher scores (Top20%) were stratified as hypoxia high whereas samples with lower scores (Bottom 20%) were stratified as hypoxia low following which gene expression levels for PRMTs was checked. This description has also been added to the materials and methods section of the manuscript.

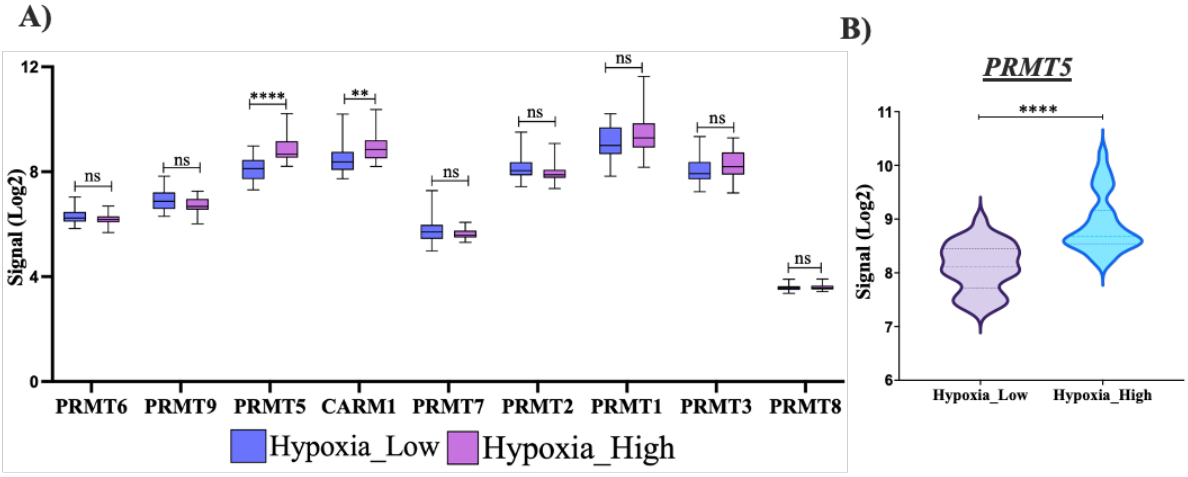

Rebuttal Figure 1. A) *Gene expression levels of all PRMTs in breast cancer patient microarray data stratified as hypoxia high or hypoxia low.* ***B)*** *Gene expression levels of PRMT5 in breast cancer patient microarray data stratified as hypoxia high or hypoxia low.*

2. I have major concerns for the H4R3me2 and H3R8me2s WB and ChIP data generated in this study as it is well known that these histone marks are expressed at very low abundance and that antibodies are not well characterised. The full WB for 1E need to be included in supplementary information and controls (GSK591) such as those shown in Figure 3C but using a lower dose of GSK591 (<1uM) as 5uM is very high. Likewise, inclusion of PRMT5 knockdown in H4R3me2s and H3R8me2s ChIP (Figure 5B) are important controls to draw conclusions.
Author Response: We thank the reviewer for raising this concern regarding the immunoblotting and ChIP data. The full blots for figure Fig 1E and 3C have been included here as well as (Rebuttal Fig 2A-C) in the supplementary document as Supplementary

Fiure1A,1B and Supplementary Figure 3D. We initially performed all our experiments using 5µM GSK591 as reported in Chiang et al., 2017 for breast cancer cells.Other studies such as Sachamitr et al.,2021 and Mai Hoang et al., 2024have reported the use of 1µM GSK591.Therefore, taking these factors into consideration and also as suggested by the reviewer, we performed immunoblotting of H3R8me2s/H4R3me2s after treating cells with 1µM GSK591. We observed a reduction in the levels of PRMT5 mediated H3R8me2s and H4R3me2s upon 1µM GSK591treatment under hypoxia (Rebuttal Fig 2D). As suggested, we have performed ChIP qPCR assay upon PRMT5 knockdown under hypoxia and observed that upon PRMT5 knockdown , PRMT5 mediated histone modifications H3R8me2s and H4R3me2s are not enriched at the TCF3 ICR region (Rebuttal Fig 2E).

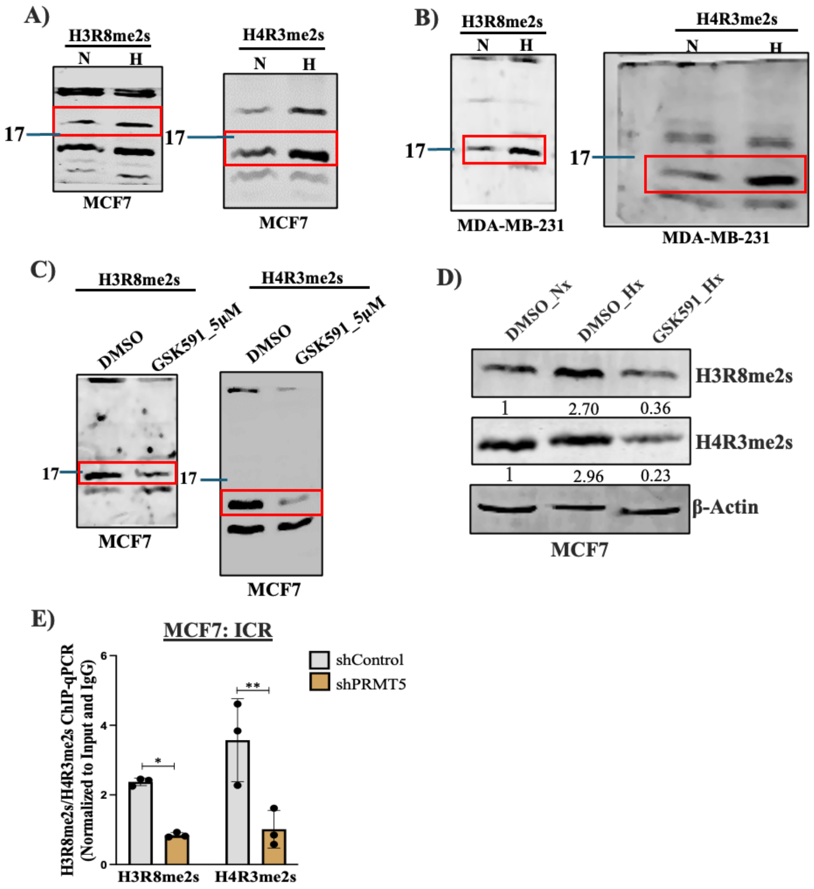

***Rebuttal Figure 2. A-B)** Raw immunoblots of figures 1E. **C)** Raw immunoblots of figure 3C. **D)** Immunoblot for H3R8me2s and H4R3me2s in MCF7 cells treated with 1µM GSK591 under hypoxia. **E)** H3R8me2s and H4R3me2s ChIP-qPCR upon PRMT5 knockdown under hypoxia in MCF7 cells.*

3. Since PRMT5 has already been associated with EMT-associated gene changes in normoxic conditions, can the authors compare the extent in changes in CDH1, CDH2, VIM and SNAI1 transcripts after PRMT5 knockdown/inhibition in hypoxic and normoxic conditions (Figure 3A, 3D).

Author Response: We thank the reviewer for the query regarding the expression status of EMT markers under normoxic conditions upon PRMT5 knockdown/inhibition. As per our observation, PRMT5 expression is considerably lesser in normoxia compared to hypoxia in breast cancer cell lines. It is known that EMT and invasion happen in normoxic conditions as well. However, hypoxia is known to enhance these characteristic features and we suggest that PRMT5 is enhancing these abilities under hypoxia and ablation of PRMT5 or its activity substantially reduces EMT and invasion under hypoxia. As suggested, we performed shRNA mediated knockdown of PRMT5 and also inhibited its activity using GSK591 inhibitor in

normoxic conditions and observed that these perturbations do not significantly affect the levels of CDH1, CDH2, VIM and SNAI1 transcripts in breast cancer cells (Rebuttal Fig 3A-B).

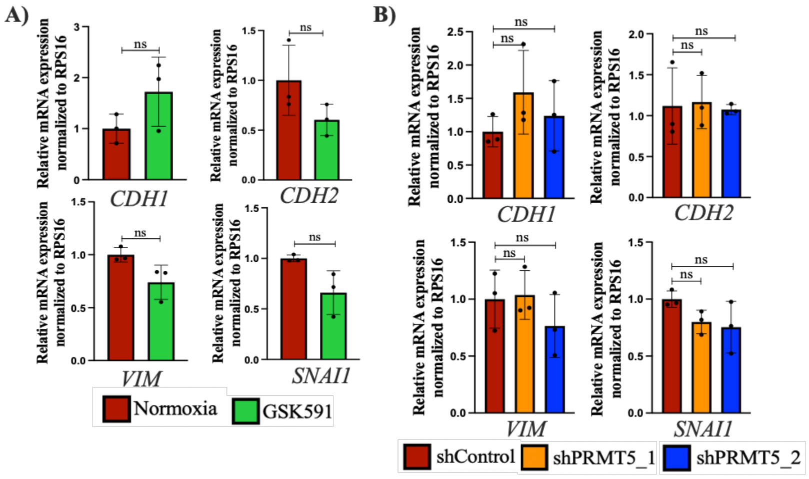

Rebuttal Figure 3. A) *qRT PCR of EMT markers upon GSK591 treatment under normoxia in MCF7 cells.* ***B)*** *qRT PCR of EMT markers upon PRMT5 knockdown under normoxia in MCF7 cells.*

4. Likewise, does knockdown of CTCF affect PRMT5 levels in normoxic conditions (Figure 2D). How can the authors be sure that the effects of CTCF KD on PRMT5 expression is not due to global disruption of gene expression given the insulator role for CTCF?

Author Response: We thank the reviewer for raising this important query regarding the role of CTCF. We performed CTCF knockdown in normoxic conditions and observed that there is no change in PRMT5 protein expression (Rebuttal Fig 4A) indicating that CTCF mediated regulation of PRMT5 happens in a hypoxia specific manner.

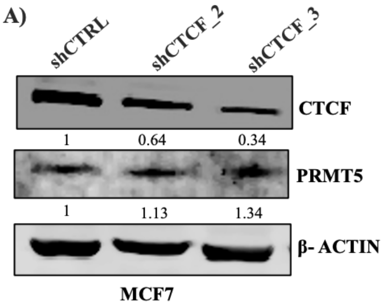

Rebuttal Figure 4. A) *Immunoblot showing PRMT5 levels upon CTCF knockdown under normoxic condition in MCF7 cells.*

5. The authors conclude because GSK591 changes the expression of EMT genes (Figure 3D/E) that "PRMT5-mediated histone modifications were involved in regulation of EMT induction after hypoxia". Given that GSK591 will affect the methylation of splicing factors and other proteins involved in transcription in addition to histones, this statement cannot be made and is misleading.

Author Response: We thank the reviewer for highlighting the statement on the role of PRMT5 histone modifications affecting EMT under hypoxia. In this study, we observed that knocking down PRMT5 under hypoxia inhibited EMT induction and invasion. This observation clearly suggested that PRMT5 has a definitive role in regulation of EMT under hypoxia. In addition, when we treated the breast cancer cells with PRMT5 activity inhibitor GSK591, we observed the same affect in corroboration with PRMT5 knockdown under hypoxia including a reduction

in the PRMT5 mediated symmetric arginine dimethylation marks on histones H3 and H4. This was further suggestive of the fact that curbing PRMT5 activity was sufficient to inhibit EMT induction and invasion. In this context, our intention was to project that loss in PRMT5 mediated histone modifications might be **one of the major causes** leading to deregulated EMT and invasion under hypoxia. As per reviewer's suggestion, the statement has been appropriately modified as; "Together, these observations suggested that PRMT5-mediated histone modifications (H3R8me2s/H4R3me2s) **could be one of the major players involved in regulation of EMT induction under hypoxia**" in the manuscript highlighting the fact that the observations made strongly suggests that PRMT5 mediated histone methylation could be one of the ways in which it could regulate EMT and invasion under hypoxia.
Additionally, using PRMT5 inhibitor and dCas9-PRMT5 system we show that PRMT5 mediated histone methylation indeed eventually regulates EMT and invasion under hypoxia.

6. ΔPSI is normally set to a 10% change. The data should be re-analysed using these thresholds as 3% change is very low. Sashimi plots for TCF3, ZEB2 and ZBTB38 should be included to show quantitatively the change in exon usage.

Author Response: We thank the reviewer for raising this important point regarding RNA-seq data analysis. We reanalyzed the RNA-seq data and we observe the TCF3 MXE splicing event to be significant (FDR<0.05) having ΔPSI >10. Since, our intended event is an MXE event , we now performed our subsequent analysis using genes listed as MXE events showing significant changes (FDR< 0.05 and ΔPSI >10). In lieu with our previous observations our GO analysis showed genes undergoing significant MXE splicing changes upon PRMT5 knockdown under hypoxia are majorly involved in regulation of EMT and apical junction. When we overlapped the MXE events showing significant changes (FDR< 0.05 and ΔPSI >10) with a list of EMT transcription factors as done before (Fig 4D), we found two overlapping genes; TCF3 and FOXM1, between the two sets. We have included the sashimi plots for TCF3 and FOXM1 showing differential splicing event (Rebuttal fig 5).

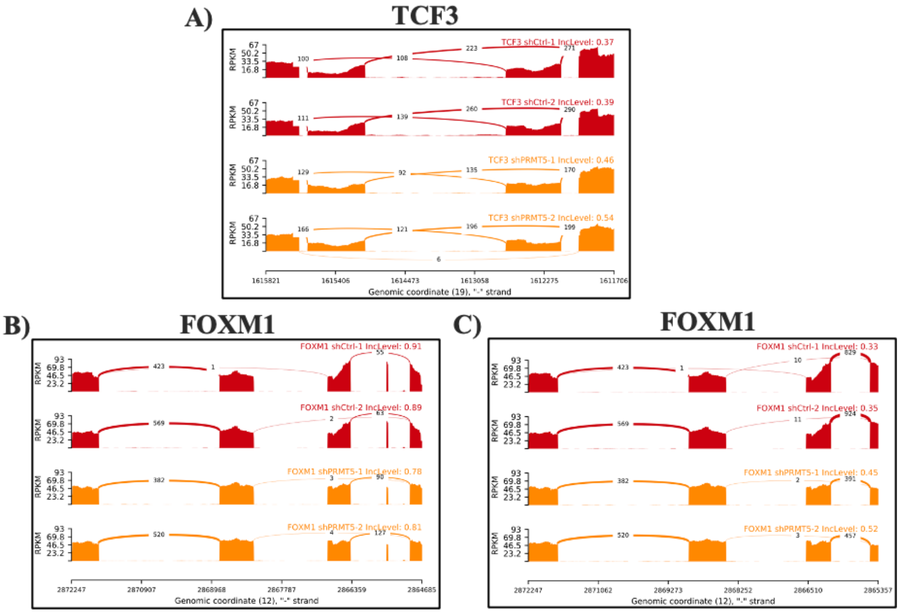

***Rebuttal Figure 5. A)** Sashimi plot showing differential exon usage of TCF3 exons 18A and 18B. B-C) Sashimi plot showing FOXM1 splicing event.*

7. Figure 4: There is no indication the number of significant spliced events (ΔPSI), rather just the classification of events. This needs to be included, along with the list of genes present in the highlighted GO categories.

Author Response: We thank the reviewer for the suggestions regarding the RNA seq analysis and gene ontology analysis. As suggested, we have included the number of significantly spliced events (Line no. 273, Page 9) (6298, FDR<0.05 and |PSI|>10%) in the manuscript. Also, the list of genes present in the highlighted GO categories has been included in the supplementary 1 excel sheet which has been provided as the supplementary attachment file.

8. Figure 4I is not controlled, a control siRNA should be included.
Author Response: We thank the reviewer for addressing this issue. We have now included a shControl for this experiment (Rebuttal Fig 6A) and the figure has been updated with the new graph in the manuscript as well.

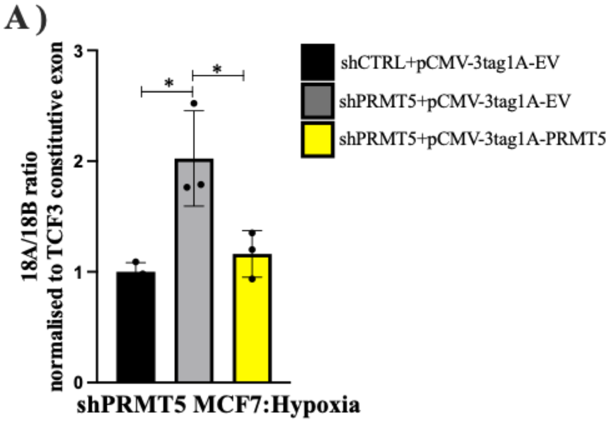

Rebuttal Fig 6. A) *qRT-PCR showing change in TCF3 splicing upon over expression of PRMT5 in PRMT5 depleted cells under hypoxia in MCF7 cells.*

9. Figure 5A that depicts the position of the ICR should be amended to include the length of the ICR and it position from slice junctions.
Author Response: We thank the reviewer for the suggestion regarding the changes in Figure 5. As suggested, We have appropriately modified the figure to include the length of the ICR.

10. Again, a conceptual jump made in the text that is not supported by the data: "We observed a significant decrease in 18A/18B ratio upon targeting dCAS9PRMT5 to the TCF3 locus (Figure 5E) indicating that local histone R dimethylation cataslyed by PRMT5 at the TCF3 splicing locus is sufficient to alter the alternative splicing pattern of TCF3". I agree that hypoxia and dCAS9PRMT5 increases histone methylation at the ICR, however the link to 18A/B isoform switch is at best correlative. **Mutation of H3/H4 at the ICR would be required to support histone methylation regulating splicing changes**. In line with this, the authors state that "We also observed that abrogation of the histone marks is sufficient to inhibit DNMT3A recruitment and DNA methylation at the TCF3 ICR region" (text after signpost for figure 5H), however I fail to see this data. If the authors are suggesting that GSK591 treatment is evidence to support this then I do not agree given that all PRMT5 methylation will be inhibited. There is also no evidence that PRMT5-mediated histone methylation impairs RNA PolII binding as again the authors are using GSK591 (Figure 6A), nor that PTBP1 recruitment is dependent on PRMT5-histone methylation (Figure 6D).
Author Response: We thank the reviewer for identifying this conceptual jump.
PRMT5 mediated histone modifications are post translational modifications which we observe to be enriched at the TCF3 ICR region under hypoxia. Since these are PTMs, it is not possible for us to specifically mutate them at the given locus. Also, perturbation of core histone genes (H3 or H4) at genetic level will have genome wide implications and would compromise the entire genome architecture thereby making locus specific interpretation infeasible. Therefore using an inhibitor such as GSK591 enables us to specifically inhibit PRMT5 activity indicating

how PRMT5 mediated symmetric dimethylation of histones H3R8 and H4R3 are essential in triggering the cascade of events leading to the regulation of TCF3 splicing. Additionally knocking down PRMT5 also would result in effects that are pleotropic like in case of using the inhibitor. Keeping these views in mind we chose to use the PRMT5 specific inhibitor GSK591 for our analysis. As shown in Figure 3, we observed that when we inhibited PRMT5 activity, PRMT5 mediated histone methylation was also downregulated. Since our aim was to see how these PRMT5 mediated histone marks regulate TCF3 splicing, we used the GSK591 inhibitor and performed all the downstream analysis.

However, since GSK591 could have pleiotropic affects as rightly pointed out by the reviewer, we now resorted to the dCas9-PRMT5 approach which is more specific and a direct way of showing that PRMT5 mediated histone methylation events at ICR are important for the splicing event under hypoxia. We made use of the dCas9-PRMT5 construct in PRMT5 knockdown cells to target the ICR locus under hypoxia. Since endogenous PRMT5 is knocked down, it allows us to track dCas9-PRMT5 mediated histone modifications specifically.

In our revised manuscript, we performed a series of downstream assays including ChIP, MeDIP and PAR-CLIP qPCRs to assess if we can recapitulate our previous observations of PRMT5 mediated regulation of TCF3 splicing. We observed that targeting the ICR locus using dCas9-PRMT5 led to enrichment of DNMT3A occupancy (Rebuttal Fig 7A) at the ICR region which further led to an increase in DNA methylation at the ICR locus (Rebuttal Fig 7B). Further, we also observed an increase in MeCP2 occupancy (Rebuttal Fig 7C) and RNA pol2 occupancy at the ICR (Rebuttal Fig 7D) when targeted using the dCas9-PRMT5 construct under hypoxia. Finally, our PAR-CLIP qPCR assay revealed that PTBP1 binding was enriched at the ICR on TCF3 RNA (Rebuttal Fig 7E) upon reintroduction of PRMT5 mediated histone methylation marks using the dCas9-PRMT5 construct.

In addition to our observations made using PRMT5 inhibitor GSK591 previously, we now show using dCas9-PRMT5 system that PRMT5 mediated histone methylation at the TCF3-ICR region is important for alternative splicing regulation under hypoxia. This epigenetic modifier system is locus specific which thereby corroborates with our hypothesis that PRMT5 regulates TCF3 alternative splicing via its ability to methylate histones. Put together these observations indicate that PRMT5 mediated symmetric histone dimethylation regulates TCF3 splicing under hypoxia in breast cancer cells.

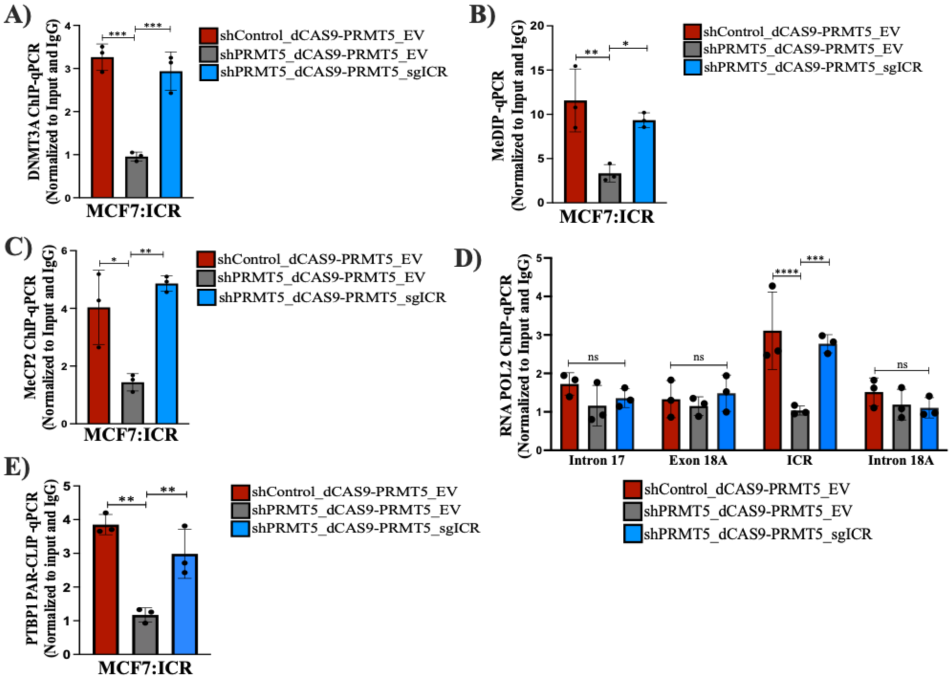

Rebuttal Fig 7. A) *DNMT3A ChIP-qPCR at TCF3-ICR region upon treatment with dCAS9-PRMT5 construct under hypoxia in shPRMT5 MCF7 cells.* ***B)*** *MeDIP-qPCR at TCF3-ICR region upon treatment with dCAS9-PRMT5 construct under hypoxia in shPRMT5 MCF7 cells.* ***C)*** *MeCP2 ChIP-qPCR at TCF3-ICR region upon treatment with dCAS9-PRMT5 construct under hypoxia in shPRMT5 MCF7 cells.* ***D)*** *RNA POL2 ChIP-qPCR at different positions along the TCF3 splicing locus upon treatment with dCAS9-PRMT5 construct under hypoxia in shPRMT5 MCF7 cells.* ***E)*** *PTBP1 PAR-CLIP-qPCR at TCF3-ICR region upon treatment with dCAS9-PRMT5 construct under hypoxia in shPRMT5 MCF7 cells.*

11. The RNA PolII ChIP experiments (Figure 6A, B) are only showing binding of RNA PolII at that locus and thus I struggle to see how the data supports alterations in RNA PolII elongation rates. As such, there is no data supporting that "MeCP2 binding leads to slower RNA PolII elongation rates".

Author Response: We thank the reviewer for highlighting this point. As rightly mentioned by the reviewer, RNA pol II ChIP experiments show enrichment of Pol II binding at a given locus. However, in previous reports, RNA pol II enrichment has been shown in comparison to other regions in the gene to indicate that RNA pol II was relatively slower at a specific locus compared to others (Brody et al., 2011; Shukla et al., 2011; Singh et al., 2017). Therefore in the revised version of our manuscript we have included a comparative analysis wherein we checked for RNA pol II occupancy at different regions preceding ICR region and downstream of the ICR region to establish the difference in RNA pol II occupancy at different sites which is a reflection of varying kinetics of RNA pol II elongation. We find that RNA pol II occupancy is enriched only at the ICR region under hypoxia and goes down when treated with GSK591 (Rebuttal Fig 8A). Similarly, upon *MECP2* knockdown we observed that RNA pol II enrichment significantly goes down specifically at the ICR region (Rebuttal Fig 8B) indicating that MeCP2 is essential for regulating RNA pol II kinetics under hypoxia. These observations are strongly suggestive of the change in RNA pol II kinetics at the ICR region which eventually regulates TCF3 splicing under hypoxia. We have also made these changes in the manuscript appropriately (Line 551-555) highlighting that higher RNA pol II occupancy is an indication of slower kinetics.

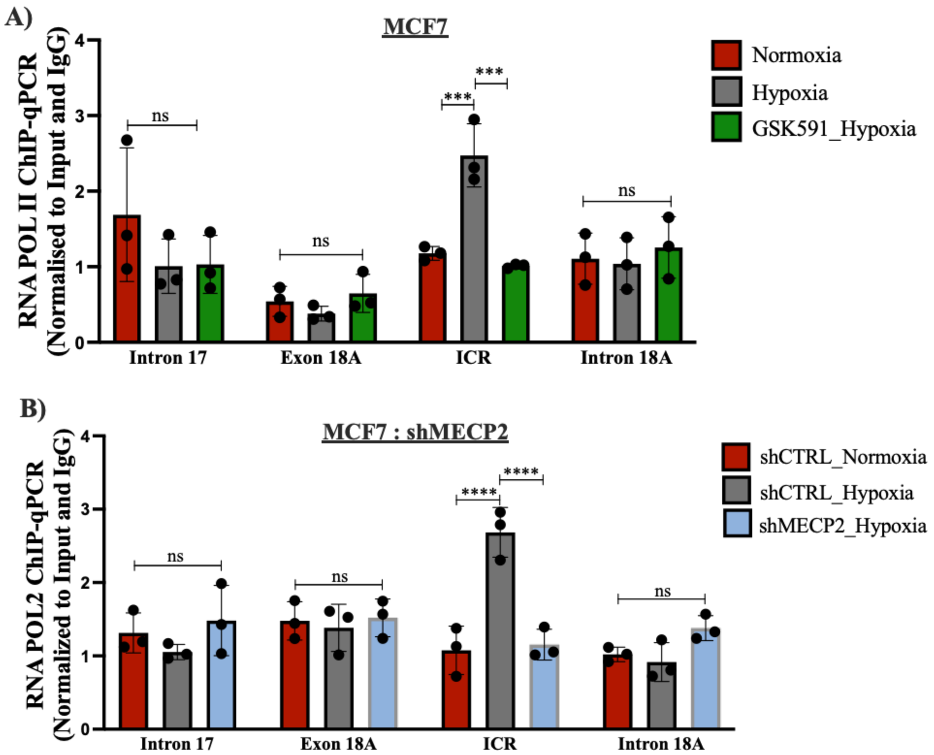

Rebuttal Fig 8. A) *RNA POL2 ChIP-qPCR at different positions along the TCF3 splicing locus upon treatment with GSK591 under hypoxia in MCF7 cells.* ***B)*** *RNA POL2 ChIP-qPCR at different positions along the TCF3 splicing locus under hypoxia in shMECP2 MCF7 cells.*

12. Data in Figure 6F is not convincing.

Author Response: We thank the reviewer for their valuable feedback on this data. We have repeated the immunoblotting experiment and we observed that TCF3-18B isoform overexpression led to decrease in E-Cad expression in MCF7 cells.

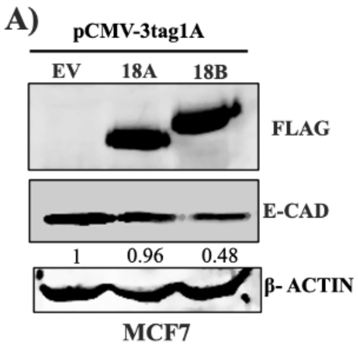

Rebuttal Fig 9. A) *Immunoblot showing E-Cad levels upon over expression of TCF3-18A and TCF3-18B isoforms in MCF7 cells.*

Minor corrections:

1. Antibodies need to be stated if used for ChIP, IHC-F or WB and what dilution was used.

Author Response: We have included these details in the supplementary table.

2. No indication of how long GSK591 is added to cell culture media before analysis.

Author Response: We have mentioned the time of GSK591 treatment in the materials and methods section under the subheading Cell culture

Reviewer #2: Within the manuscript "PRMT5 mediated histone methylation regulates alternative splicing of TCF3 via MECP2-PTBP1 to promote EMT in breast cancer hypoxia" by Mutnuru et al., the authors find that the arginine methylatransferase PRMT5 is upregulated under hypoxic conditions, dependent on displacement of CTCF. The elevation of PRMT5, in turn, impacts the expression of EMT related proteins, in general elevating their expression and regulating alternative splicing events. In particular, the alternative splicing of TCF3, as controlled by PRMT5, is studied is some detail.

Some impressive results are found within the manuscript. For example, that targeting dCAS9-PRMT5 to the TCF locus leads to alternative splicing is quite novel. This is a robust approach and informs of the importance of PRMT5 activity in regulating gene splicing. The team also found that knockdown of PRMT5 leads to localized loss of DNA methylation, strengthening a relationship between PRMT5 and DNA methylation. That the alternative spliced variant of TCF3 (18B) has enhanced abilities to promote matrigel invasion is also quite interesting.

A principal conclusion of the paper is that PRMT5 plays a unique role under conditions of hypoxia. However, many controls under normoxic conditions are missing, weakening this point. For each set of experiments carried our under hypoxic conditions, a set carried out under normoxic should be included as a control.

Author Response: We deeply appreciate the reviewer for giving us a very insightful and valuable feedback and for the positive remarks on our current study. We thank the reviewer for highlighting the weak link in our study addressing which has significantly helped us in drawing more meaningful conclusions. In our revised study we have tried to address the issue with respect to normoxic controls being necessary for drawing major conclusions. As suggested by the reviewer, we have conducted a series of experiments in the normoxic condition as well, strengthening our hypothesis that PRMT5 regulates EMT and invasion of breast cancer cells under hypoxia.

Major points:

1. Many critical "normxic" controls are missing. This is especially true for experiments carried out under hypoxic conditions, where normoxic controls are frequently missing. It is impossible to conclude that certain PRMT5 activities are specific to hypoxic conditions if normoxic controls have not been considered.

Missing controls include:
i) Figure 2I. dCAS9 targeting CTCF binding site under hypoxic conditions lead to increased DNA methylation.
- This experiment should be carried out under normoxic conditions as a control.
Author Response: We thank the reviewer for this valuable suggestion. As suggested, we targeted the CTCF binding site under normoxia using the dCas9-DNMT3A construct and we observed that there is no change in PRMT5 expression at protein level (Rebuttal Fig 10A). Also, our MeDIP qPCR and CTCF ChIP qPCR analysis upon dCAS9-DMT3A treatment under normoxia showed no significant upregulation in DNA methylation or CTCF binding at the CTCF binding site of the PRMT5 promoter (Rebuttal Fig 10B-C) indicating that CTCF mediated regulation of PRMT5 is hypoxia specific.

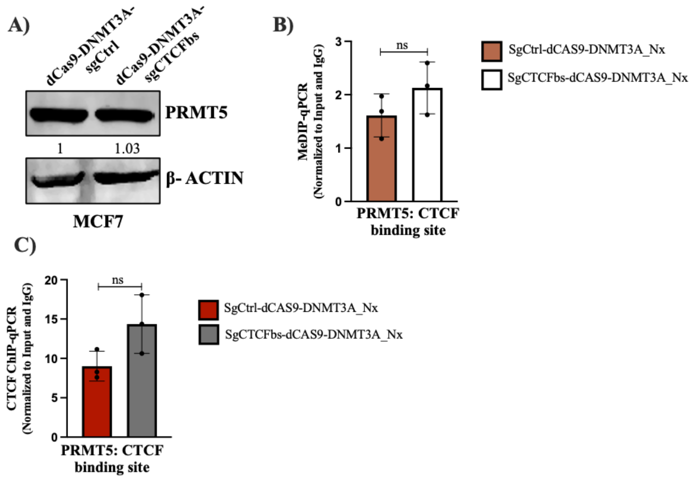

Rebuttal Fig 10. A) *Immunoblot showing PRMT5 levels upon treatment with dCas9-DNMT3A construct targeting CTCF binding site of PRMT5 promoter under normoxia in MCF7 cells.* ***B)*** *MeDIP-qPCR showing DNA methylation levels at the CTCF binding site of PRMT5 promoter upon treatment with dCas9-DNMT3A construct under normoxia in MCF7 cells.* ***C)*** *CTCF ChIP-qPCR showing CTCF occupancy levels at the CTCF binding site of PRMT5 promoter upon treatment with dCas9-DNMT3A construct under normoxia in MCF7 cells.*

ii) Figure 3F. The impact of shPRMT5 on viability under normoxic conditions should be examined as a control. PRMT5 is known to be important for viability in general. Is there a difference between normoxic and hypoxic conditions ?

iii) Similarly to pint (ii), is there a difference in response of 5µM GSK591 under hypoxia versus normoxic conditions.

Author Response: We thank the reviewer for raising this very important aspect.

We performed cell viability assay upon knockdown of PRMT5 and inhibition of PRMT5 activity using GSK591. We observed no change in viability of cells upon PRMT5 depletion or inhibition in both normoxia and hypoxic conditions in MCF7 breast cancer cells (Rebuttal Fig 11A-B).

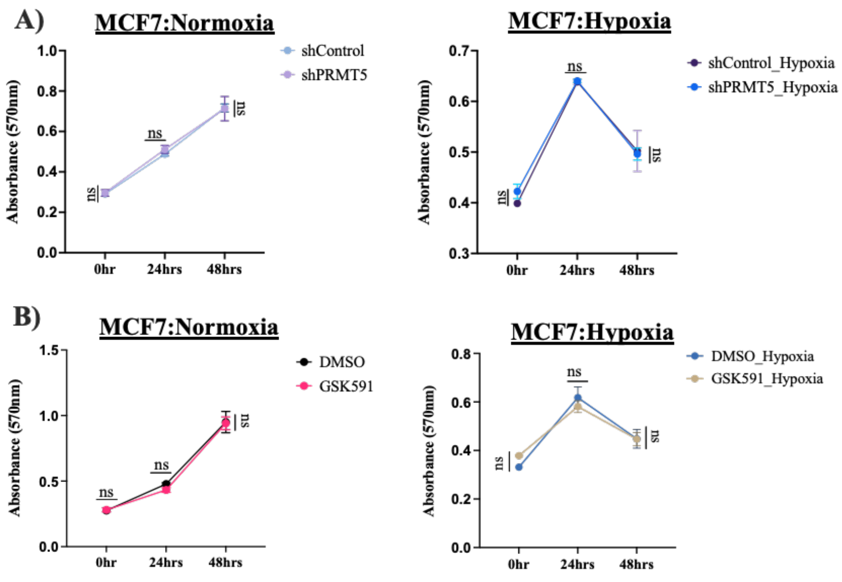

Rebuttal Fig 11. A) *Cell viability assay upon PRMT5 knockdown under normoxic and hypoxic conditions in MCF7 cells* ***B)*** *Cell viability assay upon PRMT5 activity inhibition using GSK591 under normoxic and hypoxic conditions in MCF7 cells.*

iv) Hongshan Chen et al., Oncogene 2016 showed that PRMT5 knockdown under basal conditions reduces invasion. How is the current data showing viability of PRMT5 knockdown novel?
-It is unclear that there are differences in viability or invasion between PRMT5 knockdown under normoxic or hypoxic conditions. This needs to be explored.
Author Response: We thank the reviewer for highlighting this important aspect regarding invasion assay. We knocked down PRMT5 or inhibited its activity under hypoxia using specific PRMT5 inhibitor and performed **Matrigel invasion assays** under hypoxia to highlight the fact that **PRMT5 is essential for hypoxia induced invasion.** Although Hongshan Chen et al., Oncogene, report that PRMT5 reduces cell invasion in A549 cells, we report a novel role of PRMT5 mediated regulation of cell invasion under hypoxia in breast cancer model. Additionally, we performed invasion assay upon knockdown of PRMT5 in normoxic condition and we observed no significant change in cell invasion (Rebuttal Fig 12A). This is in corroboration with our observation that PRMT5 knockdown did not result in a significant difference in expression of EMT factors (Rebuttal Fig 12B-C).

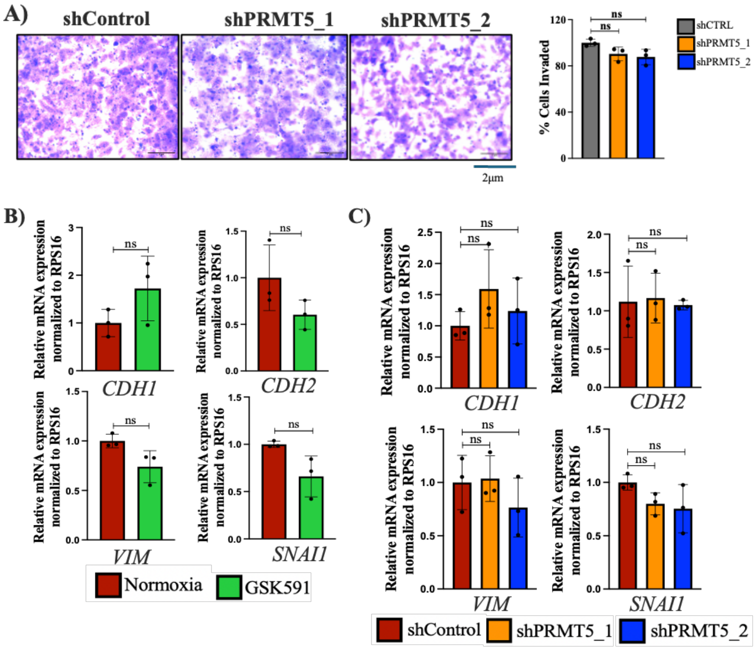

Rebuttal Fig 12. *A) Matrigel invasion assay and its quantification upon PRMT5 knockdown under normoxic condition in MCF7 cells.* ***B)*** *qRT PCR of EMT markers upon GSK591 treatment under normoxia in MCF7 cells.* ***C)*** *qRT PCR of EMT markers upon PRMT5 knockdown under normoxia in MCF7 cells.*

v) That TCF3 splicing is controlled by DNMT and PRMT5 under hypoxic conditions requires further controls. The knockdown of these enzymes should be carried out under normoxic conditions as a comparison. Likewise, shRNA-mediated knockdown of PTBP1 and its impacts should be compared under hypoxic and normoxic conditions.
Author Response: We thank the reviewer for highlighting the lack of normoxic control in case of DNMT3A and PTBP1. As suggested we performed DNMT3A and PTBP1 knockdown under normoxic conditions and verified TCF3 splicing changes. Our splicing analysis revealed no significant change in exon 18A exclusion upon knockdown of DNMT3A or PTBP1 under normoxic condition in MCF7 cells (Rebuttal Fig 13A-D).

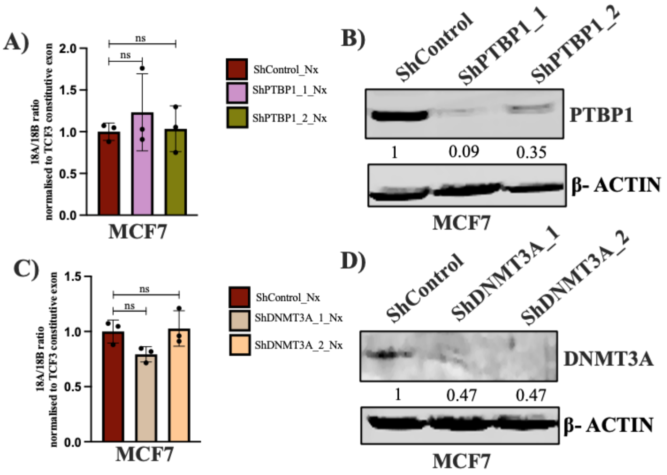

***Rebuttal Fig 13. A)** qRT PCR showing TCF3-18A/18B ratio upon PTBP1 knockdown in MCF7 cells in normoxia. **B)** Immunoblot showing knockdown of PTBP1 in MCF7 cells. **C)** qRT PCR showing TCF3-18A/18B ratio upon DNMT3A knockdown in MCF7 cells in normoxia. **D)** Immunoblot showing knockdown of DNMT3A in MCF7 cells*

2. Other important controls are missing.

i) In figure 1E it is shown that histone Arginine methylation increases under hypoxic conditions. An important control is to assess histone non-arginine methylation, such as H3K9me3, and H3K4me3. Do all methylation events change? Or is this specific to arginine methylation?

Author Response: We thank the reviewer for raising this very important query. We performed immunoblotting assay for H3K9me3 and H3K4me3 marks and observed that there is a global increase in both of these marks under hypoxia. However, there was no difference in the expression of these marks when we knocked down PRMT5 under hypoxia (Rebuttal Fig 14A) indicating that an increase in these specific histone marks is no way associated with PRMT5. Our observation thus far suggests that there could be an increase in several methylation events under hypoxia in addition to histone arginine methylation, however, H3K9me3 and H3K4me3 events are not affected by the expression of PRMT5.

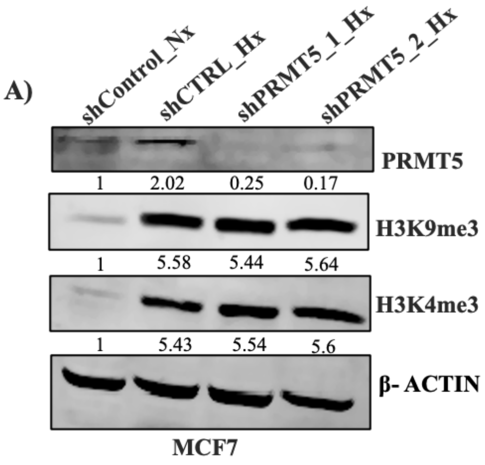

***Rebuttal Fig 14. A)** Immunoblot showing H3K9me3 and H3K4me3 levels in MCF7 cells in normoxia, hypoxia and PRMT5 knockdown condition in hypoxia, in MCF7 cells.*

ii) Figure 2F. Me-DIP controls are missing. Other CTCF-bound promoters should be examined as well as non-CTCF bound promoters.

Author Response: We thank the reviewer for highlighting the case of lack of control for the MeDIP experiment. As per our previous report (Kakani et al., 2024), we observed that CTCF binding increases and DNA methylation levels decrease under hypoxia at the COL5A1 promoter. Therefore, we checked for DNA methylation levels and CTCF occupancy at COL5A1 promoter under hypoxia as a positive control (Rebuttal Fig 15A-B). Additionally, we checked for DNA methylation and CTCF binding levels at YEATS2 promoter. Our ChIP-seq data suggests that YEATS2 promoter lacks a CTCF binding site (Rebuttal Fig 15C). Therefore we classified this region to be a non-CTCF binding promoter region. We observed that there is no significant difference in CTCF occupancy or DNA methylation levels at the YEATS2 promoter under hypoxia (Rebuttal Fig 15D-E).

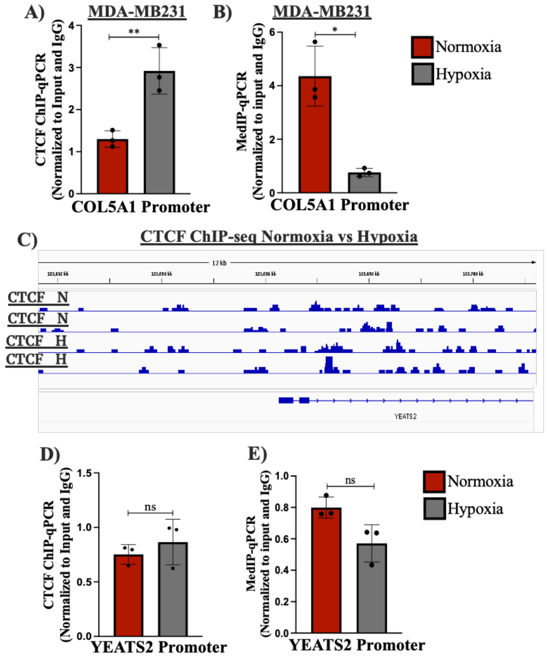

Rebuttal Fig 15. A) *CTCF ChIP-qPCR showing CTCF occupancy at COL5A1 promoter.* ***B)*** *MeDIP qPCR showing DNA methylation at COL5A1 promoter.* ***C)*** *CTCF ChIP-seq data on IGV showing no significant CTCF peaks at YEATS2 promoter.* ***D)*** *CTCF ChIP-qPCR showing CTCF occupancy at YEATS2 promoter.* ***E)*** *MeDIP qPCR showing DNA methylation at YEATS2 promoter.*

iii) Figure 6a. For ChIP-qPCR it is important to use primers targeting non-transcribed regions of the genome as negative controls. This is standard.

Author Response: We thank the reviewer for pointing this missing negative control. We identified a non-transcribed region at Chromosome 8: 49635882-49635981. We observe that there is no difference in RNA pol2 occupancy at this region.

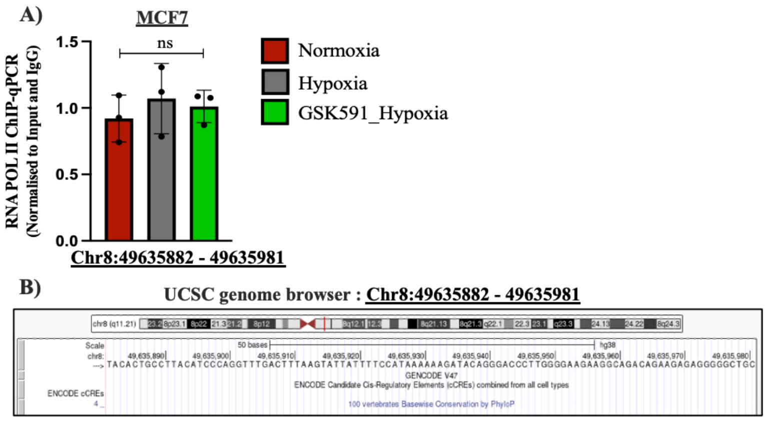

Rebuttal Fig 16. *A) RNA Pol2 ChIP-qPCR showing RNA Pol2 occupancy at a non-transcribing region,* ***Chr8:49635882 - 49635981****.* ***B)*** *Snippet from UCSC genome browser showing the* ***Chr8:49635882 - 49635981*** *region.*

3. The authors claim that PRMT5 regulates EMT-related factors via splicing events. However, Lei Huang et al, Nat Comm 2022 demonstrate that PRMT5 regulates EMT factors, especially TCF3, via translational regulation.
- How can these differences be resolved?

Author Response: Lei Huang et al, Nat Comm 2022 show that PRMT5 methylates AKT post translationally to regulate EMT and metastasis. However, there is no mention of how PRMT5 regulates TCF3 in this study. Also, there is no data suggesting that PRMT5 regulates EMT by regulating the process of translation.
However, a recent report by Gillespe et al., Oncogene, 2024 has shown that PRMT5 mediated changes in alternative splicing of DNA repair genes can influence chemoresistance. This is partially in support of our observation that PRMT5 mediated alternative splicing indeed regulates important tumorigenic events in our case that being EMT and invasion under hypoxia.

4. A major shortcoming of the paper is that alternative splicing on the level of protein expression has not been examined.
Can the alternative splicing of TCF under hypoxic conditions be confirmed by Western blotting or other proteomic approaches ?
Many studies support splicing variants under various conditions but it is rarely proven on the level of protein expression.
A Western blot of TCF using an antibody recognizing an antigen outside the spliced region under hypoxic and normoxic conditions would be insightful.

Author Response: We thank the reviewer for highlighting the importance of confirming the TCF3 splicing on protein level. As suggested, we made use of an antibody which detects the common region of both TCF3-18A (E12) and TCF3-18B (E47) isoforms. We performed immunoblotting and we observed that the E12 isoform was downregulated under hypoxia and correspondingly the E47 isoform was upregulated under hypoxia in MCF7 cells (Rebuttal Fig 17A). This observation additionally validates the functional importance of TCF3 alternative splicing under hypoxia in breast cancer cells.

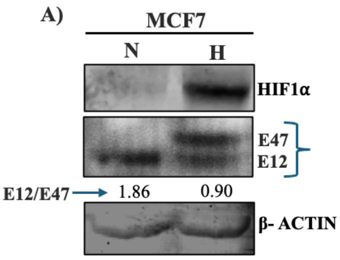

__Rebuttal Fig 17.__ A) Immunoblot showing the expression of E12 and E47 isoforms in MCF7 cells, under hypoxia.

5. The data shown in Figure 1A are challenging to interpret. It appears that all of the PRMTs remain fairly constant across both normoxic and hypoxic conditions. Only PRMT 12 seems to undergo a change in mRNA levels. Can these data be combined to a single graph showing the normoxic to Hypoxic ratio?

Author Response: We thank the reviewer for pointing out this discrepancy. We have now modified the heat map in a way that is easy to interpret.

A)

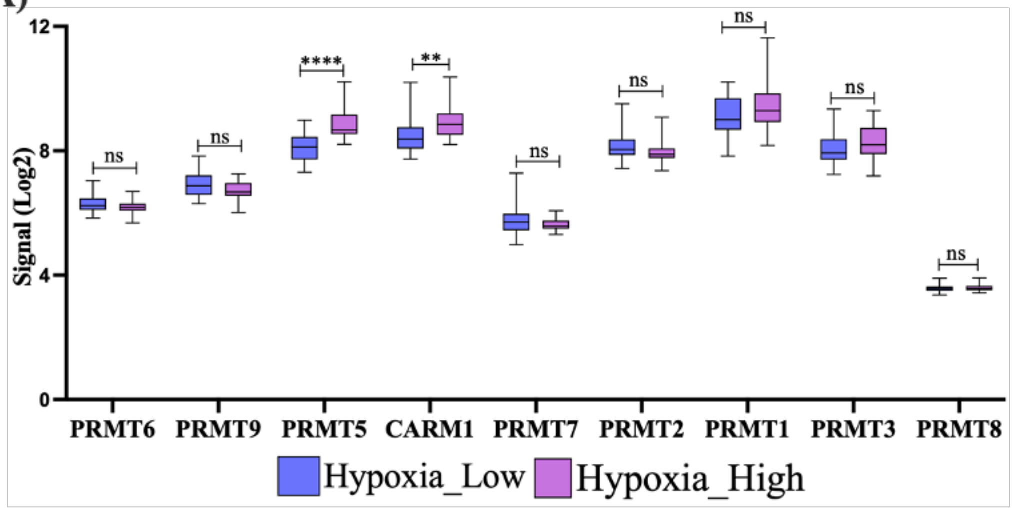

__Rebuttal Fig 18.__ A) Gene expression levels of all PRMTs in breast cancer patient microarray data stratified as hypoxia high or hypoxia low.

What does "significantly upregulated mean"? What is the level of upregulation quantified? However, the conclusion that PRMT5 is upregulated under hypoxic conditions compared to normoxic seems to be amiss.

Author Response: We thank the reviewer for highlighting this discrepancy. We quantified the level of change and indicated the values below the blot to highlight the change in expression of PRMT5 under hypoxic conditions. We observed this value to be significant and hence we used the term, "significantly upregulated." As suggested, we have included the quantification of the blots here which indicates that the upregulation of PRMT5 is indeed statistically significant.

A)

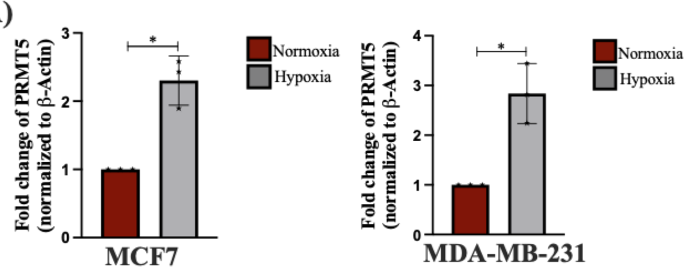

__Rebuttal Fig 19.__ A) Quantification of immunoblots representing PRMT5 upregulation under hypoxia in both MCF7 and MDA-MB-231 cells.

Reviewer #3: This paper aims to identify mechanisms through which PRMT5 influences hypoxia induce alternative splicing. A detailed series of experiments are performed demonstrating (1) That CTCF induces PRMT5 expression through a DNA methylation sensitive promoter element, (2) PRMT5 controls features of EMT in breast cancer cell lines, (3) PRMT5 regulates alternative splicing of TCF3 via DNA methylation dependent recruitment of a complex of factors (symmetric arginine demethylation, DNMT3A, MeCP2) resulting in RNA polII pausing and PTBP1 recruitment to a intronic conserved region (IRC). While hypoxia, PRMT5 and TCF3 splicing are known to be involved in EMT, this study nicely links these factors and provides another example demonstrating a link between histone modifications and their impact of alternative splicing.

Author Response: We are immensely thankful to the reviewer for the words of appreciation and a very insightful feedback on the work undertaken in this study. In our revised manuscript, we have tried to address some interesting questions with respect to HnrnpH1 and the important role of the ICR region in regulating alternative splicing of TCF3. We believe that these additions have added great value to our study and have helped us in strengthening our previously made observations.

Major comments

(1) This focus of regulator activity for TCF3 splicing appears to be the ICR, a region previously shown to be regulated be bound by PTBP1 (PMID: 31391218). In this paper Yamazaki et al propose hnRNP H1 levels to be a major determinant of TCF3 splicing outcome. Do the levels of hnRNP H1 vary upon hypoxia and contribute to TCF3 splicing?

Author Response: We thank the reviewer for highlighting this very important aspect in regulation of TCF3 splicing under hypoxia. We performed immunoblotting analysis to check HnRNPH1 levels and observed that there was a reduction in protein expression of HnRNPH1under hypoxia (Rebuttal Fig 20A). We speculate that reduced HnRNPH1 levels assist in exclusion of Exon18A under hypoxia. As mentioned in (Yamazaki et al., 2019), reduced HnRNPH1 levels promote Exon18B inclusion which is consistent with our observations. Under hypoxia, PRMT5 mediated histone methylation subsequently recruits PTBP1 to the ICR region that eventually represses 18A inclusion under hypoxia. In normoxic condition or in the absence of PRMT5, PTBP1 fails to bind to ICR which allows HnRNPH1 to supress 18B inclusion thereby promoting 18A inclusion.

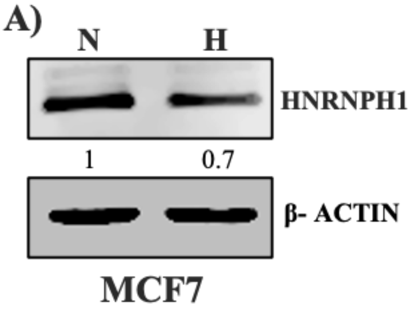

Rebuttal Fig 20. A) *Immunoblot showing HnRNPH1 levels under normoxia and hypoxia conditions in MCF7 cells.*

The majority of inferences are drawn for ChIP or CLIP qPCR analysis of the ICR region. Deletion of the genomic ICR or mutation of its sites of DNA methylation would provide stronger evidence of a link between hypoxia, PRMT5 and downstream mechanisms involved in TCF3 splicing.

Author Response: We thank the reviewer for raising this important aspect regarding the importance of the ICR region in regulation of TCF3 splicing. As suggested we performed

CRISPR/Cas9 based genome editing and successfully deleted a part of the endogenous ICR region of the TCF3 gene in MCF7 cells. We designed sgRNAs to selectively delete the region which we have reported to play an important role in regulation of TCF3 splicing via PRMT5 under hypoxia including the sites required for DNA methylation and MeCP2 binding. Genomic DNA was isolated and the edits generated were confirmed using sanger sequencing (Rebuttal Fig 21A-B). Further, we treated these ICR mutant cells under hypoxia and performed qRT-PCR analysis to check the change in 18A/18B ratio upon deletion of the regulatory region of ICR. We observed that the 18A/18B ratio increased upon mutation of ICR region under hypoxia (Rebuttal Fig 21C) indicating that the ICR is an integral regulatory region leveraged by PRMT5 to govern TCF3 splicing outcome. These observations further strengthen our hypothesis that regulation at the TCF3-ICR region mediated by PRMT5 is crucial in dictating the alternative splicing of TCF3 under hypoxia.

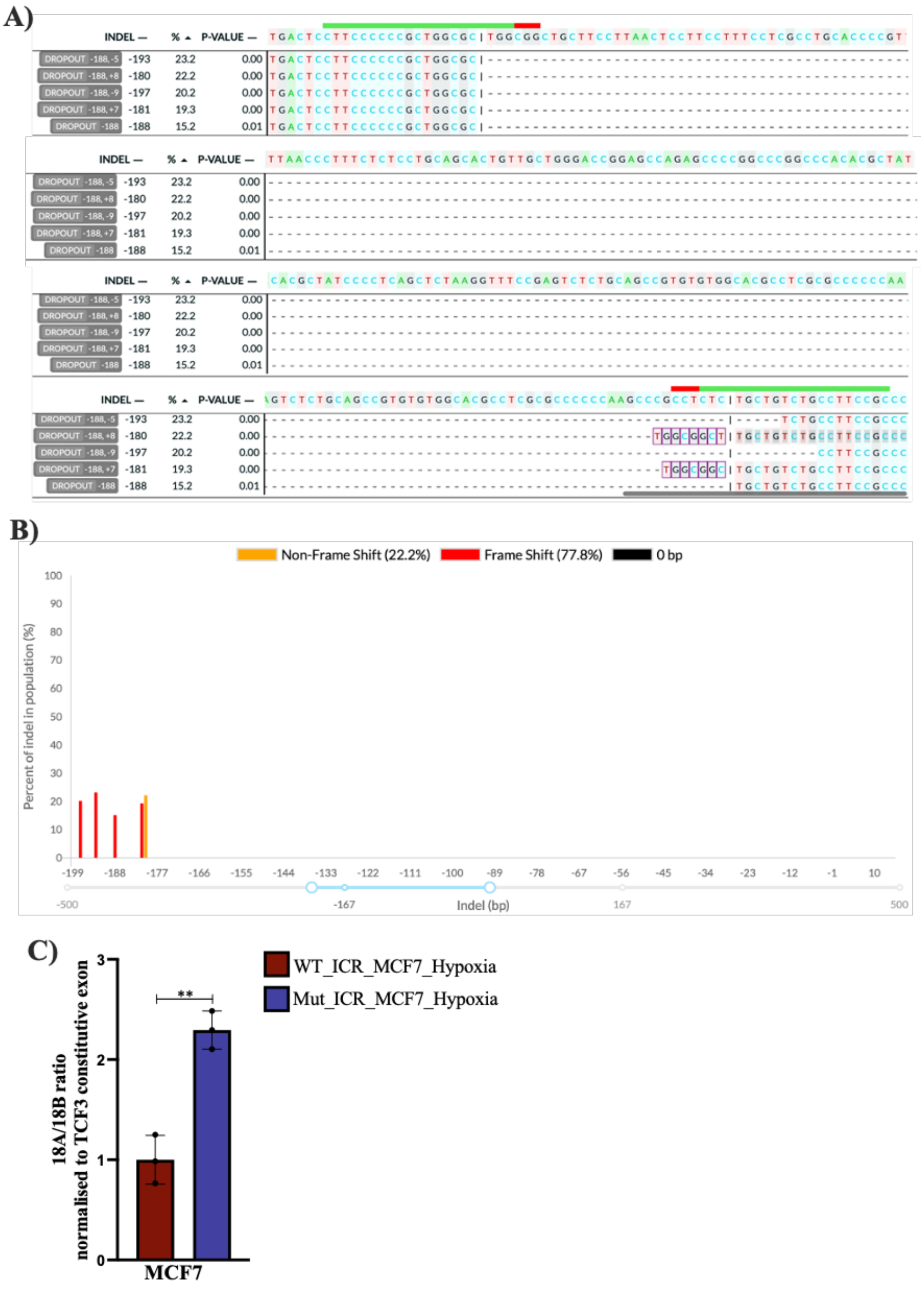

Rebuttal Fig 21. A) *DECODR map showing deletion of regulatory TCF3 ICR region in MCF7 cells.* ***B)*** *DECODR plot showing position of Indels post CRISPR/Cas9 treatment.* ***C)***

qRT-PCR showing change in exon18A/18B ratio under hypoxia upon deletion of regulatory ICR region in MCF7 cells.

(2) Two cell lines are used and treated as equivalent EMT models although are very different in their properties. MCF-7 cells are epithelial while MDA-MB-231 cells are mesenchymal and are at opposing ends of the EMT spectrum. Does hypoxia induce EMT features within MCF-7 cells, allowing them to become more invasive?

Author Response: It is correct that MCF7 and MDA-MB-231 are different in terms of their molecular properties. However, our previous reports have demonstrated that MCF7 shows enhanced EMT and invasive features under hypoxia (Ahuja et al., 2020; Dhamdhere et al., 2025; Kakani et al., 2024). Therefore we resorted to MCF7 as one of our model systems to understand EMT and invasion under hypoxia.

What is the relative amount of E47 to E12 within each cell line and how do they differ between cell lines under normoxia and hypoxia? Measurements of splicing in the paper are performed with qPCR and therefore differences in the levels of individual isoforms are difficult to assess. Does PRMT5 manipulation induce a large shift in percent spliced in (PSI) from E12 to E47 TCF3 splicing consistent with potential roles for this splice change in influencing EMT?

Author Response: We thank the reviewer for rising this query regarding TCF3 isoform expression. We quantified the expression of both TCF3-18A and TCF3-18B exons individually in both MCF7 and MDA-MB-231 cell lines. In corroboration with our findings in this study we observe that TCF3-18A isoform expression decreases under hypoxia with a concomitant increase in TCF3-18B isoform under hypoxia in both MCF7 and MDA-MB-231 cell lines (Rebuttal Fig 22A). Also, our RNA-seq analysis reveals significant difference (FDR <0.05) in PSI between Exon18A and Exon 18B upon PRMT5 knockdown under hypoxia indicating that PRMT5 manipulation indeed induces a significant shift in splicing of the TCF3 gene under hypoxia (Rebuttal Fig 22B).

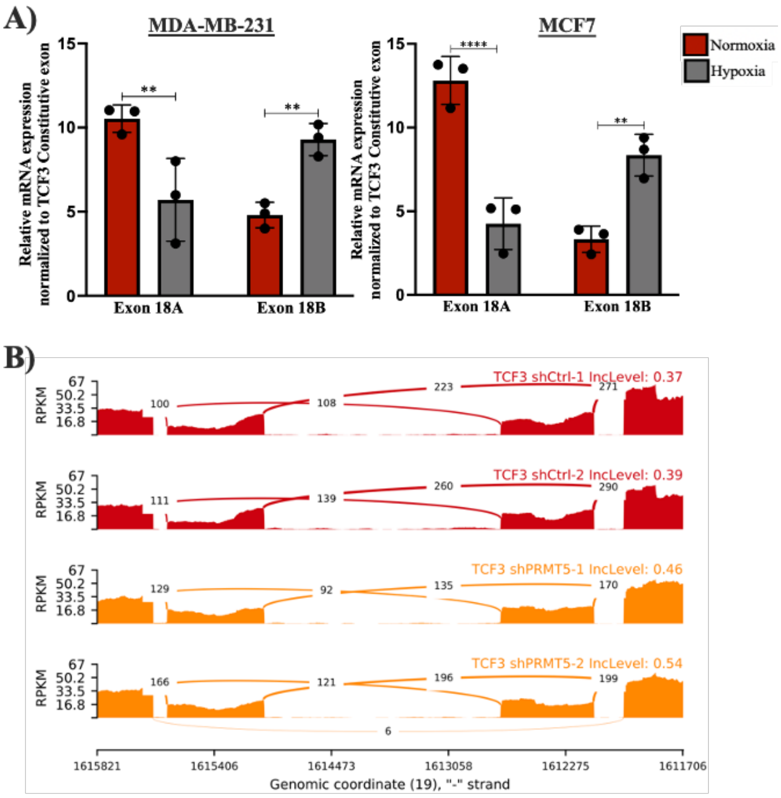

Rebuttal Fig 22. A) *Quantification of transcript levels of both TCF3-18A and TCF3-18B isoforms in MCF7 and MDA-MB231 cells in normoxic vs hypoxic condition.* ***B)*** *Sashimi plot showing. Change in TCF3 splicing event in PRMT5 knockdown condition under hypoxia.*

Minor comments

(1) The TCF3 splicing outcome is listed as a skipped exon event but is actually a mutually exclusive splicing event.

Author Response: We thank the reviewer for highlighting this aspect regarding TCF3 splicing event. We reanalyzed our RNA-seq data and have now listed TCF3 splicing as a mutually exclusive exon event.

(2) Fig 4F could be moved to Supplementary.

Author Response: We thank the reviewer for this suggestion. As suggested, we have shifted figure 4F to supplementary as Supplementary Figure 5F.

(3) Although not investigated, the authors could discuss the potential differential functions of E47 and E12 with regard to their impact on EMT.

Author Response: We thank the reviewer for giving this important comment on the variable roles of E47 and E12 isoforms. As reported by different studies, the E2A gene undergoes an alternative splicing event to produce two isoforms E12 and E47 (Yamazaki et al., 2018). Both these isoforms are class I bHLH transcription factors which bind to E box regions and regulate transcription (Massari & Murre, 2000). As a result of the alternative splicing the two isoforms harbour a slightly different bHLH DNA binding domain in the protein sequence. This apparent difference in the bHLH domain can vary the degree of DNA binding ability of both these isoforms ((Murre et al., 1989). Previous reports have suggested that E47 has a greater binding affinity to DNA compared to the E12 isoform (Murre et al., 1989). The E12 isoform has been reported to be indispensable during development whereas E47 isoform has specific roles in development (Beck et al., 2009; Benezra et al., 1990). In the context of breast cancer the E47 isoform has been reported to promote stemness and tumor invasion (López-Menéndez et al., 2021; Slyper et al., 2012). Taken together, we speculate that E12 and E47 might bind differently to DNA in normoxic condition compared to hypoxia respectively. It would be interesting to evaluate if epigenetic modifications have any role in determining the binding specificities of these proteins.

References

Ahuja, N., Ashok, C., Natua, S., Pant, D., Cherian, A., Pandkar, M. R., Yadav, P., Narayanan S.S., V., Mishra, J., Samaiya, A., & Shukla, S. (2020). Hypoxia-induced TGF-β–RBFOX2–ESRP1 axis regulates human MENA alternative splicing and promotes EMT in breast cancer. *NAR Cancer*, *2*(3), zcaa021. https://doi.org/10.1093/narcan/zcaa021

Beck, K., Peak, M. M., Ota, T., Nemazee, D., & Murre, C. (2009). Distinct roles for E12 and E47 in B cell specification and the sequential rearrangement of immunoglobulin light chain loci. *Journal of Experimental Medicine*, *206*(10), 2271–2284. https://doi.org/10.1084/jem.20090756

Benezra, R., Davis, R. L., Lockshon, D., Turner, D. L., & Weintraub, H. (1990). The protein Id: A negative regulator of helix-loop-helix DNA binding proteins. *Cell*, *61*(1), 49–59. https://doi.org/10.1016/0092-8674(90)90214-Y

Brody, Y., Neufeld, N., Bieberstein, N., Causse, S. Z., Böhnlein, E. M., Neugebauer, K. M., Darzacq, X., & Shav-Tal, Y. (2011). The in vivo kinetics of RNA polymerase II elongation during co-transcriptional splicing. *PLoS Biology*, *9*(1). https://doi.org/10.1371/journal.pbio.1000573

Chiang, K., Zielinska, A. E., Shaaban, A. M., Sanchez-Bailon, M. P., Jarrold, J., Clarke, T. L., Zhang, J., Francis, A., Jones, L. J., Smith, S., Barbash, O., Guccione, E., Farnie, G.,

Smalley, M. J., & Davies, C. C. (2017). PRMT5 Is a Critical Regulator of Breast Cancer Stem Cell Function via Histone Methylation and FOXP1 Expression. *Cell Reports*, *21*(12), 3498–3513. https://doi.org/https://doi.org/10.1016/j.celrep.2017.11.096

Dhamdhere, S. G., Bansal, A., Singh, P., Kakani, P., Agrawal, S., Samaiya, A., & Shukla, S. (2025). Hypoxia-induced ATF3 escalates breast cancer invasion by increasing collagen deposition via P4HA1. *Cell Death & Disease*, *16*(1), 142. https://doi.org/10.1038/s41419-025-07461-y

Kakani, P., Dhamdhere, S. G., Pant, D., Joshi, R., Mishra, J., Samaiya, A., & Shukla, S. (2024). Hypoxia-induced CTCF promotes EMT in breast cancer. *Cell Reports*, *43*(7). https://doi.org/10.1016/j.celrep.2024.114367

López-Menéndez, C., Vázquez-Naharro, A., Santos, V., Dubus, P., Santamaría, P. G., Martínez-Ramírez, Á., Portillo, F., Moreno-Bueno, G., Faraldo, M. M., & Cano, A. (2021). E2A Modulates Stemness, Metastasis, and Therapeutic Resistance of Breast Cancer. *Cancer Research*, *81*(17), 4529–4544. https://doi.org/10.1158/0008-5472.CAN-20-2685

Mai Hoang, P., Torre, D., Jaynes, P., Ho, J., Mohammed, K., Alvstad, E., Yee Lam, W., Khanchandani, V., Min Lee, J., Min Clarissa Toh, C., Xue Lee, R., Anbuselvan, A., Lee, S., Sebra, R. P., Walsh, M. J., Marazzi, I., Kappei, D., Guccione, E., & Jeyasekharan, A. D. (2024). A PRMT5-ZNF326 axis mediates innate immune activation upon replication stress. In *Sci. Adv* (Vol. 10). https://www.science.org

Massari, M. E., & Murre, C. (2000). Helix-Loop-Helix Proteins: Regulators of Transcription in Eucaryotic Organisms. *Molecular and Cellular Biology*, *20*(2), 429–440. https://doi.org/10.1128/mcb.20.2.429-440.2000

Murre, C., Mccaw, P. S., & Baltimore, D. (1989). A New DNA Binding and Dimerization Motif in lmmunoglobulin Enhancer Binding, daughterless, MyoD, a&i myc Proteins. In *Cell* (Vol. 56).

Sachamitr, P., Ho, J. C., Ciamponi, F. E., Ba-Alawi, W., Coutinho, F. J., Guilhamon, P., Kushida, M. M., Cavalli, F. M. G., Lee, L., Rastegar, N., Vu, V., Sánchez-Osuna, M., Coulombe-Huntington, J., Kanshin, E., Whetstone, H., Durand, M., Thibault, P., Hart, K., Mangos, M., … Dirks, P. B. (2021). PRMT5 inhibition disrupts splicing and stemness in glioblastoma. *Nature Communications*, *12*(1), 979. https://doi.org/10.1038/s41467-021-21204-5

Shukla, S., Kavak, E., Gregory, M., Imashimizu, M., Shutinoski, B., Kashlev, M., Oberdoerffer, P., Sandberg, R., & Oberdoerffer, S. (2011). CTCF-promoted RNA polymerase II pausing links DNA methylation to splicing. *Nature*, *479*(7371), 74–79. https://doi.org/10.1038/nature10442

Singh, S., Narayanan, S. P., Biswas, K., Gupta, A., Ahuja, N., Yadav, S., Panday, R. K., Samaiya, A., Sharan, S. K., & Shukla, S. (2017). Intragenic DNA methylation and BORIS-mediated cancer-specific splicing contribute to the Warburg effect. *Proceedings of the National Academy of Sciences*, *114*(43), 11440–11445. https://doi.org/10.1073/pnas.1708447114

Slyper, M., Shahar, A., Bar-Ziv, A., Granit, R. Z., Hamburger, T., Maly, B., Peretz, T., & Ben-Porath, I. (2012). Control of Breast Cancer Growth and Initiation by the Stem Cell–Associated Transcription Factor TCF3. *Cancer Research*, *72*(21), 5613–5624. https://doi.org/10.1158/0008-5472.CAN-12-0119

Yamazaki, T., Liu, L., Lazarev, D., Al-Zain, A., Fomin, V., Yeung, P. L., Chambers, S. M., Lu, C. W., Studer, L., & Manley, J. L. (2018). TCF3 alternative splicing controlled by hnRNP H/F regulates E-cadherin expression and hESC pluripotency. *Genes and Development*, *32*(17–18), 1161–1174. https://doi.org/10.1101/gad.316984.118

Yamazaki, T., Liu, L., & Manley, J. L. (2019). *TCF3 mutually exclusive alternative splicing is controlled by long-range cooperative actions between hnRNPH1 and PTBP1.* https://doi.org/10.1261/rna
